# Classical and Modified Ketogenic Diets for Children and Young People With Drug‐Resistant Epilepsy: A Reflection of International Dietetic Practice and Best Practice Recommendations for Dietitians

**DOI:** 10.1111/jhn.70129

**Published:** 2025-10-12

**Authors:** Natasha E. Schoeler, Victoria J. Whiteley, Monica Guglielmetti, Lenycia de Cassya Lopes Neri, Robyn Blackford, Charlene Tan‐Smith, Nicole Aylward, Katie Barwick, Maria Eugenia Caballero, Sylwia Gudej‐Rosa, Wan‐Ling Huang, Meredith Johnson, Christine Ong Bee Keow, Sarianne Madsen, Kath Megaw, Theresa Nyembezi Njobvu, Heidi Pfeifer, Sian Phillips, Elles van der Louw, the Best Practice Recommendations Advisory Group, on behalf of the Ketogenic Dietitians Research Network, Christine Au Yeung, Tessa Bollard, Ramona DeAmicis, Diana Lehner‐Gulotta, Nicole Mills, Candy Richardson, Christi Sports, Lisa Vanatta, Kristel Van de Kerckhove, Rocio Viollaz, Sze Man Wong

**Affiliations:** ^1^ UCL Great Ormond Street Institute of Child Health London UK; ^2^ Great Ormond Street Hospital for Children London UK; ^3^ Royal Manchester Children's Hospital Manchester UK; ^4^ University of Pavia Pavia Italy; ^5^ Lurie Children's Hospital of Chicago Chicago Illinois USA; ^6^ Christchurch Hospital Christchurch New Zealand; ^7^ HSC Winnipeg Shared Health Winnipeg Manitoba Canada; ^8^ Children's Health Queensland Queensland Australia; ^9^ Hospital de Pediatria J P Garrahan Buenos Aires Argentina; ^10^ Institute of Mother and Child Warsaw Poland; ^11^ Linkou Chang Gung Memorial Hospital Taoyuan City Taiwan; ^12^ Children's Healthcare of Atlanta Atlanta Georgia USA; ^13^ KK Women's & Children's Hospital Singapore City Singapore; ^14^ UCSF Benioff Children's Hospital San Francisco California USA; ^15^ Kath Megaw & Associates Cape Town South Africa; ^16^ University Teaching Hospitals Lusaka Zambia; ^17^ Boston Children's Hospital Boston Massachusetts USA; ^18^ Southampton Children's Hospital Southampton UK; ^19^ Erasmus University Medical Center Rotterdam The Netherlands; ^20^ Oakland Medical Center Oakland California USA; ^21^ Sydney Children's Hospital Sydney New South Wales Australia; ^22^ University of Milan Milan Italy; ^23^ UVA Health Charlottesville Virginia USA; ^24^ Cambridge University Hospitals Cambridge UK; ^25^ Duke University Hospital‐The Children's Health Center Durham North Carolina USA; ^26^ Phoenix Children's Phoenix Arizona USA; ^27^ UZ Leuven Leuven Belgium; ^28^ Hospital de niños Sor M. Ludovica La Plata Argentina

**Keywords:** classical ketogenic diet, clinical, dietetic, epilepsy, guidelines, modified ketogenic diet

## Abstract

**Introduction:**

A global need was identified for a practical, comprehensive tool to guide dietitians internationally working in ketogenic diet therapy (KDT), detailing all aspects of dietetic management. The aim of this project was to develop best practice recommendations for the dietetic management of classical and modified ketogenic diets in the management of epilepsy and neurometabolic conditions.

**Methods:**

Expert ketogenic dietitians from six continents were invited to participate as members of either a core working group or advisory group. A systematic literature review was conducted, covering all aspects of dietetic management, from patient selection to diet discontinuation. To complement this, an international survey was distributed to ketogenic dietitians currently delivering classical and modified ketogenic diets, structured around the same key themes.

**Results:**

A total of 111 dietitians responded to the survey, representing six continents. For each theme, findings from the literature were presented alongside survey responses. Recommendations were generated where ≥ 75% consensus was achieved. In areas where this threshold was not met, the most commonly reported practices were presented, acknowledging the variety of international approaches.

**Conclusion:**

These are the first international best practice recommendations specifically for ketogenic dietitians and nutrition healthcare professionals supporting children following medically advised classical and modified ketogenic diets. The recommendations are informed by both published evidence and prevailing international dietetic practice, whilst recognizing the variety in clinical delivery.

## Summary

- Classical ketogenic diets are defined as ‘ketogenic diets that are based on a ratio of grams of fat to grams of protein and carbohydrate combined, usually ranging from 2:1 to 4:1’, based on our international survey responses.
- Modified ketogenic diets are defined as ‘ketogenic diets that include a carbohydrate limit and a fat target, where protein is either unrestricted or guided by general age‐based targets, and that do not fit the definition of a classical ketogenic diet or a medium chain triglyceride ketogenic diet’, based on our international survey responses.
- This manuscript outlines the first international best practice recommendations aimed at dietitians managing children and young people with epilepsy referred for treatment with a classical or modified ketogenic diet, as part of a multi‐disciplinary team. All aspects of dietetic management are covered, from patient selection to diet discontinuation.

**Abbreviations**
BKD: Blended ketogenic diet
BMI: body mass index
CKD: classical ketogenic diet
IV: intravenous
KDRN: ketogenic dietitians research network
KDT: ketogenic diet therapy
LGIT: low glycemic index treatment
MAD: modified atkins diet
MCT: medium chain triglyceride
MKD: modified ketogenic diet

## 1 Introduction

Ketogenic diet therapy (KDT) is an umbrella term describing a group of high‐fat, low‐carbohydrate, moderate protein diets used as a management option for drug‐resistant epilepsy.

The classical ketogenic diet (CKD) is the ‘original’ form of KDT, described by Wilder in 1921 [1], and is based on a ratio of grams of fat to grams of carbohydrate and protein combined. We adopt the terminology ‘classical’, coined in 1989 [2], in keeping with the dictionary term meaning ‘traditional in style or form’ (Cambridge Dictionary), as used in the UK, mainland Europe and further afield; we do, however, acknowledge that the term ‘classic’ ketogenic diet is also used, particularly in the USA, as has been debated in recent literature [3]. To allow for a higher carbohydrate intake and/or protein without compromising ketosis, Huttenlocher later introduced a variant of KDT incorporating medium chain triglyceride (MCT) fat, with 60% of energy derived from MCTs [4]. A modified version using 30% of calories from MCT was subsequently developed to reduce gastrointestinal side effects [2].

In 2006, following a case series of six individuals with epilepsy treated with a version of the popular ‘Atkins’ diet [5], the ‘Modified Atkins Diet’ (MAD) was formally described [6]. MAD typically restricts carbohydrate to 10 g/day for the first month, encourages high fat intake, and allows unrestircted protein. More recently, the term ‘Modified Ketogenic Diet’ (MKD) has appeared in the epilepsy literature, although it was not comprehensively described until 2019 [7]. Another variation, the low‐glycemic index treatment (LGIT), is a more liberal low‐carbohydrate diet that may or may not be ketogenic [8]. LGIT typically permits 40–60 g of carabohydrate per day, all with a glycemic index < 50, with fat comprising 60%–70% and protein 20%–30% of total energy intake.

All KDT types except the CKD can come under the umbrella term of ‘modified ketogenic diets’; however, terminology remains inconsistent. For example, The Charlie Foundation refers to ‘modified keto’ as a CKD with ratios lower than 4:1, while Matthew's Friends and much of the clinical literature, align more closely with MAD and MKD definitions.

Optimal clinical management recommendations for healthcare professionals managing children with epilepsy on KDT were updated in 2018 [9]. While these include guidance relevant to dietitians, they are predominantly clinically focused and do not explore the full spectum of dietetic practice, such as calculating prescriptions or inititating or discontinuing KDT.

A clear gap was identified for a practical resource tailored to the needs of dietitians delivering KDT. Due to the discrepancy in use of the terms MKD and MAD in recent years, these KDT types were chosen to focus on first, alongside CKD, the most commonly used KDT [10].

We aimed to explore the breadth of international dietetic practice in KDT and develop best practice recommendations informed by the published iterature and the most commonly reported approaches in clinical practice.

## 2 Materials and Methods

A call for expert volunteers to contribute to the development of ‘best practice dietetic recommendations for classical and modified ketogenic diets’ was circulated via the Ketogenic Dietitians Research Network (KDRN) and ketogenic dietitians. listserv mailing list.

Sixteen dietitians were selected to form the core working group, each with a minimum of 5 years' clinical experience in KDT, including the use of CKD, MAD or MKD. Where possible, the number of dietitians from each geographical area (USA, Canada, UK and Europe, Central and South America, Middle East and Asia, Africa and Oceania) was aligned proportionally to the number of ketogenic centers in that area, based on listings from The Charlie Foundation, Matthew's Friends and The International League Against Epilepsy. In the case of over‐representation of volunteers from one country/continent, selection was based on a short written statement outlining each candidate's experience and capacity to contribute to the project. Due to overwhelming interest from certain regions, an additional ‘advisory group’ was formed to provide expert review of draft manuscripts. As with the core group, efforts were made to ensure proportional representation by geographical area, relative to the number of ketogenic centres.

### 2.1 Literature Search

A dedicated subgroup of the core working group conducted the literature review. A virtual training session was delivered and the topics and search strategy were standardized and agreed.

Each subgroup member was assigned a specific subtopic, with the aim of collating published evidence related to the dietetic management of CKDs or MKDs, ranging from initial referral to diet discontinuation. The subtopics included:

- Patient selection
- Pre‐diet preparation (dietetic and psychosocial)
- Diet prescription, including macronutrients, fluids, vitamins and minerals, enteral feeding, special dietary requirements, different age groups
- Prescribable ketogenic products
- Diet initiation
- Monitoring, including adverse effects, management of illness, psychosocial impact, adherence and tele‐healthcare
- Diet discontinuation

The following core terms were used to search the online PubMed database, last updated on 24/02/2024: (child* OR infant) AND (ketogenic OR “modified atkins”) AND (epilepsy OR seizure*), followed by additional search terms according to the subtopic (see Table S1 for full search terms). Relevant data were extracted into a structured Excel template capturing the following fields: author, year of publication, article title, study design, age of participants/cohort, type of KDT and details relevant to each subtopic. Findings for each subtopic were then summarised in bullet‐point format.

Of 530 articles retrieved from the literature search, 155 studies were included following title/abstract and full‐text screening. Of the 155, five articles addressed patient selection, 20 pre‐diet preparation, 23 diet prescription, 11 prescribable ketogenic products, 39 diet initiation, 50 diet monitoring and 12 addressed diet discontinuation.

### 2.2 Survey

A subgroup of the core working group was responsible for developing and delivering the survey. A total of 131 multiple choice questions were created, aligned with the same subtopics explored in the literature review. Each question included a free‐text or ‘other’ option to allow respondents to describe alternative practices and capture the diversity of global dietetic approaches. The survey was distributed via KDRN and. listserv using the online platform SmartSurvey™ (SmartSurvey Ltd, Tewkesbury, United Kingdom).

A total of 111 dietitians anonymously completed the survey, with the following proportion georographical representation: Europe (31%), North America (23%), South America (14%), Australia (11%), Southeast Asia (6%), East Asia (5%), Canada (4%), New Zealand (4%), Southern Africa (3%), South Asia (1%), and West Asia (1%).

Survey responses were summarized using descriptive statistics. For questions where participants could ‘Select all that apply,’ response percentages may exceed 100%. Where questions required a choice between types of KDT (e.g. for specific patient populations), only CKD and MKDs were presented as options, reflecting the specific focus of this project. All survey answers are given in Supporting material (Supporting sheet 1).

### 2.3 Writing the ‘Best Practice Recommendations’

Bullet‐point summaries from the literature review formed the basis of the main document and were presented alongside the corresponding survey results. To reflect the diversity of international dietetic practice while maintaining clarity and conciseness, survey answers selected by ≥ 5% of respondents were included. In specific cases, such as reporting on the length of time taken to discontinue KDT, all response options were presented in full. Unless otherwise stated, all percentages reported in the text refer to the proportion of respondents selecting that particular answer in the survey.

A set of core recommendations was developed for each aspect of CKD and MKD dietary management. These were drawn from either:

– Published literature (restricted to published consensus recommendations, international guidelines, systematic reviews, meta‐analyses or randomized controlled trials), or
– Survey responses that reached a consensus threshold of ≥ 75%. Where this threshold was not achieved in survey responses (e.g., with ‘Select all that apply’ questions), the most commonly selected option(s) were reported and wording carefully chosen to acknowledge the variability in dietetic practice.

The worked examples and meal planning sections were developed based on the clinical experience of the core and advisory working groups to give practical suggestions to readers. The draft manuscript and recommendations were first reviewed by the core working group. Following revisions, the updated manuscript was reviewed by the advisory group. Any points of contention were discussed and resolved via virtual meeting.

## 3 Results: International Dietetic Practice

### 3.1 Defining Classical Ketogenic Diets

The CKD is the original form of KDT, first described by Wilder in 1921 [1]. It remains the most commonly used KDT in clinical practice [10]. The diet protocol is based on a ratio of grams of fat, to grams of protein and carbohydrate, with each meal or feed carefully calculated. While the literature suggests that ratios above 1.7:1 promote ketogenesis [11], in practice, lower ratios may still be effective depending on the individual's metabolic response. In the survey, 77% of survey respondents defined CKDs as those with a fat to nonfat ratio between 2:1 to 4:1. A further 18% considered only higher ratios (3:1 to 4:1) to qualify as a CKD. Additionally, 27% defined CKDs as dietary protocols that require all foods to be weighed on a gram scale.

Based on the most common survey responses, this guideline defines CKDs as ‘ketogenic diets that are based on a ratio of grams of fat to grams of protein and carbohydrate combined, usually ranging from 2:1 to 4:1’.

### 3.2 Defining Modified Ketogenic Diets

MAD was first described as a diet in which carbohydrates are restricted to 10 g/day for the first month, fat is ‘encouraged’ and protein is unrestricted [6]. In contrast, MKDs have been defined in a variety of ways in the literature, including diets that contain approximately 80% fat [12], or ‘in which carbohydrates, protein, and fat are individualized’ [13], or where protein intake is ‘1 g/kg dosing weight using either actual or adjusted weight using a 25% adjustment factor’, with varying amounts of carbohydrate and fat goals [14]. Household measures or weighed portions for fat and carbohydrate foods may be used [7].

Survey responses highlighted variation in how MKDs (including MAD) are defined across ketogenic diet centers:

– 54% of respondents described MKDs as KDT with a defined carbohydrate limit and fat target, while allowing protein freely
– 22% also specified calorie and protein targets
– 35% of respondents reported calculating the diet ratio, though approximately half of these noted that this was used only as a dietetic guide, not a formal prescription.

Table S2 outlines the variety of features of MKDs adopted by international centers.

Based on the most common definitions in the survey, MKDs are here defined as ‘ketogenic diets that include a carbohydrate limit and a fat target, where protein is either unrestricted or guided by general age‐based targets, and that do not fit the definition of a CKD or an MCT KD’.

### 3.3 Patient Selection

The role of the multidisciplinary team (MDT) is paramount in deciding whether to accept an individual to start KDT. Ideally, all potential patients should be evaluated within a tertiary epilepsy specialist center, where medical, dietetic, and psychosocial factors can be jointly considered [15]. Typically, the medical team leads on referral and/or acceptance of patients for consideration of KDT.

All patients referred for KDT should be screened for metabolic contraindications before initiation. This includes testing serum acylcarnitine profile and/or urine organic acids or serum amino acids [9]. There is currently no definitive consensus regarding the use of KDT during pregnancy [16]. Adolescent females of child‐bearing age should be advised to exercise caution and, where pregnancy is confirmed or planned, the decision to initiate or continue KDT should be based on a careful risk‐benefit assessment. From a dietetic perspective, certain factors that *may* complicate following KDT should also be screened for and considered before accepting a referral (Table 1).

**Table 1:** Potential contraindications to ketogenic diet therapy: survey responses.

Factor	Agreement rate (% survey respondents)
Medication/treatment noncompliance	63%
Lack of family support	61%
Long‐term parenteral nutrition	52%
Difficulties with reading and/or understanding (patient or family, as appropriate)	51%
Severe picky eating	50%
Emesis	47%
Gastrointestinal disorders (gastroparesis, short bowel etc)	46%
Poor oral intake, especially fluids and/or formula	38%
Bone fragility	28%
Financial difficulties	26%
Gastro‐esophageal reflux	23%
Endocrine disorders	22%
Language difficulties (e.g. if their native language is different to that of the clinical team)	21%
History of aspiration pneumonia	16%

*Note:* None of the above are necessarily absolute contraindications to starting a medical ketogenic diet, but rather factors to consider. The risk‐benefit ratio should be considered in each individual.

#### 3.3.1 Survey Results

Table 1 outlines factors that, according to survey respondents, *may* complicate following KDT.

CKD was favoured over MKDs by respondents in the following scenarios:

- Enteral feeding (88%)
- Age < 2 years (70%)
- Clinically unstable, hence needing to get better seizure control quickly (66%)
- Family preference (58%)
- Epilepsy syndrome/type, e.g. Glucose Transporter Type 1 Deficiency Syndrome (GLUT1DS) (41%) or Pyruvate Dehydrogenase Deficiency (PDHD) (42%))

MKD was favoured by respondents over CKD in the following scenarios:

- Family preference (80%)
- Oral feeding (67%)
- Age > 12 years (56%)
- Limited health or diet literacy in the patient or family (48%)
- Very high carbohydrate intake at baseline (41%)
- Age > 2 years (30%)

For individuals with GLUT1DS, 35% of survey respondents preferred either CKD or MKDs, depending on contextual factors such as patient age, family understanding, age at diagnosis, age at diet start, mode of feeding, symptom severity, anticipated diet duration, and patient acceptance of dietary change. CKD was preferred in infants under 1 year of age. Only 6% of respondents favoured MKDs for GLUT1DS regardless of these factors. For individuals with PDHD, 42% of survey respondents preferred CKD, while 31% reported that either CKD or MKDs could be appropirate depending on individual circumstance.

### 3.4 Pre‐Diet Preparation

In addition to medical team responsibilities, such as baseline laboratory assessments, nutrition/dietetic and psychosocial preparation play a crucial role in setting the foundation for succesful KDT.

#### 3.4.1 Dietetic Assessment and Preparation

As part of the pre‐diet preparation, the dietitian should complete a comprehensive diet history. This may include a 3‐day dietary record and/or a detailed account of their usual dietary intake, including feeding method, textures tolerated, food allergies, intolerances and other special dietary requirements. Conducting a diet history not only helps to establish the patient and families commitment to KDT, but also provides invaluable insight into habitual calorie intake, eating behaviours, and personal or cultural preferences [17].

Any food allergies, intolerances or special dietary requirements should be carefully documented and considered within the KDT prescription. A thorough review of current and previously trialled formulas or foods can help avoid unnecessary dietary restrictions [18].

The dietitian should also collect baseline anthropometry measurements, including weight and length/height, and where appropriate, mid‐upper arm circumference and skinfold thickness. These measurements, along with growth history and laboratory results, such as full blood count, ferritin and vitamin D levels, support the assessment of the patient's nutritional status and requirements to inform the diet prescription [17]. It is also important to determine whether the patient is already or has previously been, under dietetic care.

Survey findings showed that over half of survey respondents recommend that patients make dietary adjustments before KDT initiation, for example by reducing simple sugars (59%), trying out high‐fat foods (49%) or trying keto foods/recipes (46%).

#### 3.4.2 Patient/Family Education and Counselling

Parental education is a critital component of KDT and plays a major role in both diet initiation and long‐term adherence. While clinical and nutritional safety form the foundation of the education, discussions should also address the broader implications of following a restrictive medical diet. These conversations help minimize potential financial, developmental and psychological burdens, and can improve family preparedness and reduce the risk of non‐adherence [19, 20, 21, 22].

The literature identifies key barriers to KDT adherence, which should be explored during education sessions:

- Time investment required for meal/feed preparation [19, 22]
- Adverse effects of KDT [19]

- Parental anxiety pre‐diet [23]
- The cost of KDT‐compatible supplements and/or specific foods [24]
- Cultural adaptability of KDT [23]
- Disruption of the home environment [23]
- Poor palatability and restrictiveness of KDT [19].

To support families effectively, clinicians should assess the family's capabilities (knowledge, skills and ability to make dietary change), their resources (food availability, support from school, etc), and their motivation to start KDT to best support adherence. Some centres may be equipped with a social worker or child life specialist who can assist but, often, the dietitian plays a central role in this process. Dietitians must also be aware of the patient/family's culture and religious, and socioeconomic background to ensure that education is delivered sensitively and appropriately [19, 20, 24].

##### 3.4.2.1 Survey Results

Survey findings revealed that 92% of respondents delivered at least one structured education session before starting KDT. Education is provided individually (77%) or via group sessions (5%); the timing of the education varies either before (31%), as part of (23%) or after (28%) the baseline visit.

Education sessions are most often led by the dietitian, but may also involve other members of the multidisciplinary team, including specialist nurses, medical doctors, dietetic assistants/support workers or clinical psychologists. The format of education varies across centres, with sessions delivered face‐to‐face, virtually, or by telephone, depending on local service models and family preference.

A wide range of topics are covered in these sessions, tailored to the individual patient and family. The most commonly covered topics, according to survey responses, are outlined in Table 2.

**Table 2:** Topics to cover in initial education sessions for patients/families: survey responses.

Topic	Agreement rate (% survey respondents)
Importance of adherence to the diet	94%
Potential adverse effects	93%
Follow‐up expectations	86%
Identifying carbohydrates, protein and fat sources	83%
Weighing/measuring of foods and/or formula	83%
Importance of hydration	81%
Other sources of carbohydrates, such as medications	80%
Supplementation	79%
Time commitment needed for ketogenic diets	77%
How to check ketone levels	69%
How to recognize symptoms of hyperketosis and hypoglycemia	68%
Potential impact on quality of life *(may be negative or positive)*	64%
Other nonfood sources of carbohydrates	60%
Potential social/personal impact of following a restricted diet when around others	59%
How to use a ketone/blood glucose monitor	59%
Developing recipes and using meal calculation tools	56%
Insurance coverage and/or availability of supplies required for monitoring and diet	55%
Plan for sick days	51%
The cost of ketogenic diet‐suitable foods	42%
Strategies to manage special occasions	41%
Physical activity with reference to bone health	10%

### 3.5 Diet Prescription

Literature review data are outlined in the below sections, according to specific macronutrients.

88% of survey respondents indicated that the dietitian is responsible for calculating the initial diet prescription. The most commonly reported factors influencing macronutrient targets were:

- Age (64%)
- Usual diet (44%)
- Epilepsy syndrome (36%)
- Medications (14%)
- Physical activity levels (13%)
- Calorie and protein requirements (6%).

**Figure 1:**
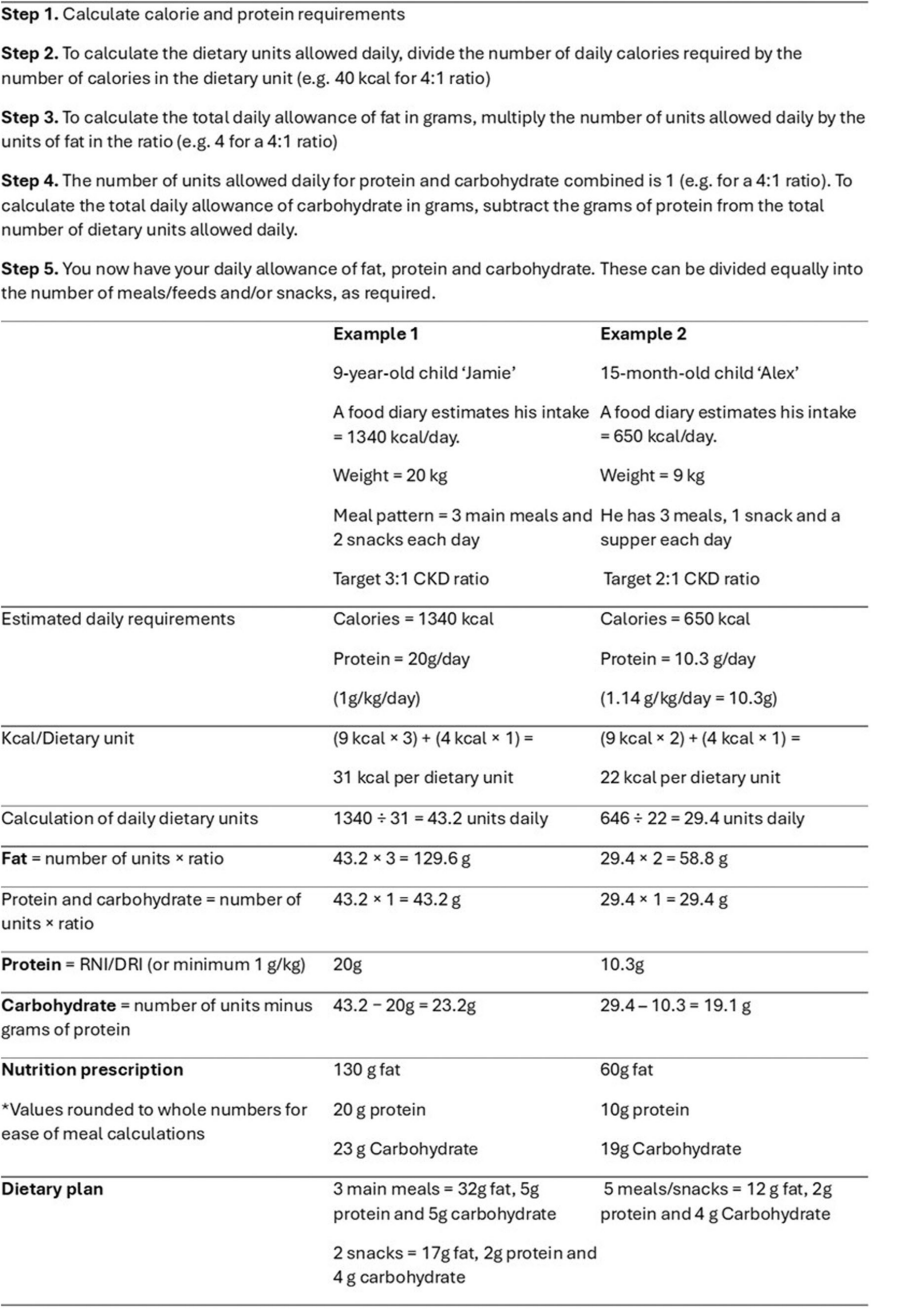
Worked example for calculating a classical ketogenic diet.

An example calculation for CKD is presented in Figure 1, based on the most commonly used macronutrient targets and prescription strategies reported in the survey. Calculations are presented using the dietary unit method, as described by Kossoff [25]. In this approach, a dietary unit represents the combined caloric contribution of fat, protein, and carbohydrate based on a specific fat‐to‐nonfat ratio. For example, in a 4:1 ratio, a dietary unit is calculated as (4 × 9 kcal) + (1 × 4 kcal) = 40 kcal per unit. This unit‐based approach allows for structured meal planning while maintaining the prescribed ketogenic ratio.

**Figure 2:**
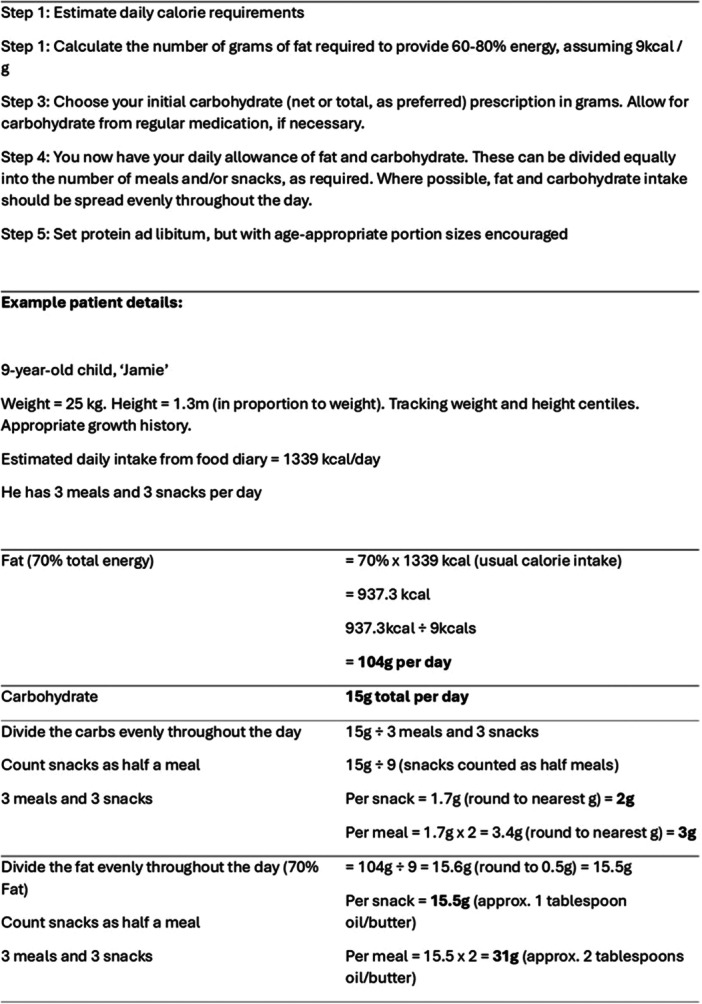
Worked example for calculating a modified ketogenic diet prescription.

MKD prescriptions are typically calculated using fixed carbohydrate limits and fat targets, with protein either unrestricted or set according to general age‐based requirements. See Figure 2 for an example calculation, based on the most commonly used macronutrient targets and prescription strategies reported in the survey.

#### 3.5.1 Energy

Historically, CKDs were prescribed with caloric restriction (80%–90% of estimated requirements) in an attempt to promote ketosis and seizure control. However, evidence suggests that energy restriction does not provide additional benefit, and may even hinder growth, particularly in younger patients or those with already compromised nutritional status. As a result, caloric restriction is no longer routinely recommended [9].

Accurate estimation of energy requirements is important when setting specific macronutrient targets, especially for CKD and more structured forms of MKD. In contrast, in many MKD protocols, energy is not explicitly prescribed to the patient or family [26, 27]; instead, carbohydrate limits and fat targets are prioritised, and energy intake is more flexible.

Energy requirements may be estimated using age and gender‐specific equations for Estimated Average Requirements (either country‐specific or from the World Health Organization [28]). For children with neurological disabilities, standard equations may not reflect true energy needs, which can be influenced by mobility, feeding difficulties, altered metabolism, and disease severity. A detailed food diary, and growth history, should be used alongside predictive equations to guide appropirate energy prescriptions.

The following percentage of survey respondents adopt the following practices for CKD prescriptions (multiple responses could be selected):

- 86% consider the patient's current energy intake
- 82% use standardised energy equations
- 71% factor in recent growth trends
- 12% still apply slight energy restriction, typically targeting 90% of estimated needs, to account for lower respiratory quotient or reduced activity levels

For MKD prescriptions:

- 78% consider the patient's current energy intake
- 77% use standard energy equations
- 65% factor in recent growth trends

#### 3.5.2 Choice of Ratio (CKD Only)

The choice of initial target ratio varies depending on factors such as age, clinical setting (e.g. critical care or outpatients) or clinical need (e.g. higher ratios may be desired sooner for optimal ketosis and clinical improvements). In practice, 35% of survey respondents start at a lower ratio and increase the ratio over time, with the aim of increasing ketosis and/or clinical efficacy. In contrast, 20% favor starting CKD at a higher ratio and then reduce the ratio over time, depending on ketone levels, clinical efficacy, or adverse effects. All meals and snacks are usually in the same diet ratio, so the relative proportions of macronutrients remain the same throughout the day.

#### 3.5.3 Fat

With CKDs, the prescribed ketogenic ratio determines the quantity in the diet. A higher ratio corresponds to a greater proportion of energy from fat. For example, in a 4:1 CKD, there are 4 g of fat for every 1 g of protein and carbohydrate combined, meaning that approximately 90% of the daily energy is derived from fat. Fat in CKDs is typically sourced from long‐chain triglyceride (LCT) sources, although MCTs may be included to enhance ketone production and allow for a slightly more liberal intake of carbohydrate or protein.

In contrast, the original MAD protocol did not prescribe a specific fat target, but fats were ‘encouraged’ [6]. MKDs have more recently been described as providing ‘~80% fat’ [12]. In the UK version of MKD, fat tends to account for 65%–80% estimated energy requirements [7].

Survey responses reflected this diversity in MKD fat prescription. One‐third of respondents prescribed 60%–70% of total energy from fat when initiating an MKD, while 23% used 70%–80%.

In terms of calculating diet prescriptions, approximately one‐third of respondents reported using a sequential method to define macronutrient targets: first estimating overall energy requirements, then setting the carbohydrate limit, followed by determining protein needs, with the remaining energy allocated to fat. This approach reflects the flexible and adaptive nature of MKD prescriptions, particularly when balancing dietary preferences, clinical goals, and patient/family capacity.

#### 3.5.4 Protein

In the CKD literature, protein intake is typically prescribed in grams/kg body weight, using Dietary Reference Intakes (DRI) or Reference Nutrient Intake (RNI) for age [29]. Individualised dietetic judgement is essential, with regular reassessment of nutritional adequacy through growth monitoring, clinical review, and biochemical parameters. While protein must be carefully calculated to maintain the ketogenic ratio, it should still meet the child's physiological requirements. Some protocols aim to meet the lowest safe level of protein intake to preserve the ketogenic ratio, whereas others prioritise ensuring at least the minimum recommended daily intake. In practice, a balance is often sought between maintaining ketosis and supporting adequate growth and nutritional status.

In contrast, the MAD generally does not restrict protein. Published studies describe protein intake as “not restricted” [30, 31] or “unlimited” [32]. Similarly, in the UK version of the MKD, protein is often not formally prescribed, although when targets are set, they typically account for 20%–25% of total energy intake [7].

Survey respondents demonstrated a range of approaches to protein prescription. In the context of CKD, nearly half of practitioners (46%) reported that they provide protein to meet the DRI or RNI as a minimum, while 54% aim to meet the full DRI. Just over half of respondents (53%) indicated that they prioritise protein over carbohydrate when calculating the macronutrient prescription. Conversely, around one‐quarter of respondents (24%) reported aiming to meet only the lowest safe intake of protein, consistent with more traditional, ratio‐driven approaches to CKD.

For MKD, practice was even more variable. Approximately 39% of respondents reported that they do not prescribe a specific number of grams of protein in the initial diet plan. Instead, protein intake is left flexible or guided by food choices. Around 30% of respondents provide guidance based on minimum daily requirements, without specifying precise targets. A smaller proportion (16%) set a maximum protein intake, typically ensuring it does not exceed 2 g per kilogram of body weight. A few respondents (5%) reported using a target range based on goal BMI, most commonly prescribing between 1.0 and 1.2 g/kg/day.

#### 3.5.5 Carbohydrate

In KDT, the term ‘net carbohydrates’ is frequently used, but defintions very internationally. In clinical practice, net carbohydrates are most commonly defined as the total cabohydrate content minus fiber, and in some cases, minus some or all sugar alcohols, which have minimal digestible carbohydrate and glycaemic affect (Table 3).

**Table 3:** Definitions of ‘net carbs’ used in ketogenic diet therapy: survey responses.

Definition of ‘net carbs’	Agreement rate (% survey respondents)
Total carbs minus fiber	40%
Total carbs minus fiber and all sugar alcohols	9%
Total carbs minus fiber and 50% sugar alcohols, except erythritol which is 10% of carbs	7%
Total carbs minus fiber and 50% sugar alcohols, except erythritol which is 10% of carbs, and minus 10% monk fruit or allulose	5%

The relevance of this distinction depends heavily on national food labelling regulations. In countries such as the United States and Canada, fibre and sugar alcohols are included in the total carbohydrate count on nutrition labels. In contrast, food labelling regulations in the United Kingdom, Europe, and Australia exclude fiber from the total carbohydrate content, and therefore the term “net carbohydrate” is less commonly used.

For clarity and consistency, all references to carbohydrate within this document refer to net carbohydrates, as defined by the prescribing clinician or local practice.

In CKDs, carbohydrate intake is typically calculated after determining the ketogenic ratio and protein requirements. The remaining caloric allowance is allocated to carbohydrate, maintaining the specified fat‐to‐nonfat ratio. The exact number of grams will therefore vary based on the energy prescription and dietary ratio used.

In MKDs, carbohydrates are generally prescribed as a fixed number of grams per day, rather that as a proportion of total energy. In the MAD literature, this allowance often begins at 10–15 g, traditionally including sugar alcohols but excluding fiber [31, 32, 33]. In the UK version of MKD, initial carbohydrate prescriptions tend to fall between 15–30 g per day, or roughtly 5%–20% estimated total energy requirements [7].

According to survey data, 72% of respondents reported specifying the daily carbohydrate allowance in their MKD prescriptions, typically adjusting for the patient's age. In contrast, 24% of respondents do not factor in age when determining the carbohydrate limit, and only 7% do not set a specific carbohydrate target in initial MKD prescriptions.

Table 4 presents the most common carbohydrate allowances used in MKD prescriptions across different age groups, with most dietitians using consistent values for both total and net carbohydrates. A more detailed breakdown of alternative allowances and respondent preferences can be found in Table S3.

**Table 4:** Carbohydrate allowances used in initial prescriptions for modified ketogenic diets: survey responses.

Age group	Carbohydrate allowances most commonly used in initial MKD prescriptions	
	Total (g)	Net (g)
< 2 years	10	10
2–5 years	10	10 or 15
6–11 years	15	15 or 20
12–18 years	15	20

##### 3.5.5.1 Carbohydrate – Other Sources

For patients receiving modified texture diets (across any type of KDT), 60% of survey respondents reported using low‐carbohydrate thickeners, such as gum‐based thickeners. 26% count starch‐based thickeners within the daily carbohydrate allowance, while 26% only count thickeners when clinically indicated ‐ for example, if ketones are suboptimal. A small proportion (5%) reported never counting either gum‐ or starch‐based thickeners in the prescription.

Medications and supplements can contain carbohydrates, particularly oral solutions, suspension, and chewable tablets, which may contribute significantly to the total carbohydrate intake. If not accounted for, this can impair ketosis or require further restriction of dietary carbohydrate.

Clinical approaches to managing carbohydrate from medications vary:

- Engage with the pharmacy team to review and change medications to low‐carbohydrate versions (typically tablets or capsules instead of oral solutions or chewable tablets) before initiating KDT
- Include the carbohydrate from medication as part of the KDT prescription (the diet plan may require recalculation if the dosages are significantly altered)
- Do not count the carbohydrate from medication and continue with the usual diet prescription, particularly if doses are small or carbohydrate is unavoidable

Survey data were not collected on which of these strategies are most commonly adopted in practice.

#### 3.5.6 Fluid

Historically, fluid prescriptions for CKD ranged from 80% to 100% of estimated daily requirements [34] and no restrictions were set for MKDs [30]. More recent guidance indicates that fluid restriction is not beneficial and may, in fact, be counterproductive [8]. Maintaining adequate hydration is essential for preventing constipation, supporting metabolic processes, and reducing the risk of nephrolithiasis, particularly in patients taking carbonic anhydrase inhibitors, such as topiramate or zonisamide.

Survey data reflected broad agreement with current recommendations. Ninety‐six percent (96%) of respondents reported prescribing fluids to meet 100% of estimated fluid requirements in CKD protocols, and 93% reported doing so for MKDs. This near‐unanimous consensus supports the shift away from historical fluid restriction practices. Fluid needs may be determined using country‐specific age‐ and weight‐based equations, or by continuing the patient's pre‐KDT fluid intake if clinically appropriate. The method of calculation varies between centres, depending on local protocol and clinician preference.

#### 3.5.7 Micronutrient Supplementation

The restricted nature of KDT significantly alters the intake of vitamins, trace minerals, and electrolytes compared to a well‐planned traditional diet [35, 36]. Micronutrient deficiencies are most commonly reported in individuals following CKDs [37, 38, 39, 40]. Documented deficiencies include thiamine, folate, pantothenic acid, calcium, phosphorus, iron, vitamin D, Vitamin C, selenium, ferritin, and magnesium, as well as carnitine (a conditionally essential amino acid, not a micronutrient). Several case reports of scurvy have been published in individuals on CKD who were not receiving adequate vitamin C supplementation [40, 41]. Selenium deficiency has also been reported in children on CKD [37], and has been associated with impaired myocardial function [42]. One study found selenium levels declined after 6 and 12 months on CKD, prompting recommendations for close monitoring [38], although it remains unclear whether selenium supplementation beyond standard multivitamin is necessary [9]. It is generally accepted that the more restrictive the ketogenic diet, the greater the risk of micronutrient deficiencies. This risk applies not only to CKDs but also to MKDs, which still impose significant limits on food variety and intake.

To prevent micronutrient deficiences, optimal clinical management recommendations are to provide a complete carbohydrate‐free multivitamin and mineral supplement for individuals on KDT. This should include, at a minimum, selenium, calcium and vitamin D [9].

If a carbohydrate‐free formulation is not available or tolerated, the carbohydrate content of an alternative supplement should be included in the overall dietary prescription. Supplementation should be aligned with age‐specific recommended daily intakes, taking into account the individual's dietary intake and any contributions from commercial ketogenic products or formula feeds.

In addition to preventing deficiencies, some micronutrients may play a role in mitigating the adverse effects of KDT. Particular attention should be given to calcium and vitamin D, as individuals on KDT can be at increased risk of acidosis and impaired bone health, including reduced bone mineral density and osteoporosis [43].

##### 3.5.7.1 Survey Results

In clinical practice, 95% of survey respondents reported being responsible for advising on micronutrient supplementation for patients on CKD. The majority (91%) routinely prescribed multivitamin and mineral supplements, either as standard practice or based on diet analysis. The most commonly prescribed specific supplements were carnitine when a deficiency was identified (52%), calcium (44%), and vitamin D (35%). Routine use of carnitine, zinc, or selenium – regardless of deficiency status – was less common, each reported by 5%–9% of respondents. Potassium citrate was prescribed prophylactically by 25% of respondents and in cases of hypercalciuria by 13%. Sodium bicarbonate was routinely supplemented by 5%, and used for the management of acidosis by 20%.

For MKD, responses were similar. 90% of respondents reported routinely prescribing multivitamin/mineral supplements, or doing so as indicated based on dietary intake. The most commonly used additional supplements were carnitine (if deficient, 42%), calcium (43%), and vitamin D (27%). A small number of practitioners (5%) noted that micronutrient supplementation for MKDs was managed by the medical team rather than the dietitian. Supplementation with potassium citrate or sodium bicarbonate was less frequent than with CKD: potassium citrate was prescribed prophylactically by 14%, in response to hypercalciuria by 13%, and sodium bicarbonate in cases of acidosis by 16%.

### 3.6 Special Dietary Requirements

With appropriate planning and professional support, most special dietary requirements can be accommodated within KDT. This includes dietary restrictions related to food allergies, intolerances, cultural or religious practices, and personal preferences such as vegetarianism or veganism.

When adapting a ketogenic diet to meet these needs, particular attention should be given to micronutrient adequacy, as food substitutions may reduce the variety or nutrient density of the diet. Vitamin and mineral supplementation should be reviewed and adjusted accordingly to prevent deficiencies.

It is important to obtain a detailed history of not only the patient's dietary requirements, but also any allergies or restrictions within the household, as these may affect food preparation, safety, and adherence to the prescribed diet.

Families may benefit from a tailored list of ingredient substitutions that are suitable for their dietary needs—for example, dairy‐free, egg‐free, or vegan alternatives. A summary of commonly used ketogenic ingredients and suggested substitutions is provided in Table S4.

#### 3.6.1 Survey Results

Survey responses confirmed that MKDs are generally preferred over CKDs when accommodating plant‐based dietary preferences. Approximately 48% of respondents reported preferring MKDs for individuals following a vegan diet, and 66% indicated a preference for MKDs for those who are vegetarian. This preference reflects the greater flexibility offered by MKDs in food choices, macronutrient distribution, and ingredient substitution.

To support the success of vegetarian or vegan KDT, respondents reported using a range of strategies. The most common was the incorporation of MCT‐enriched products, used by 60% of respondents. Commercial ketogenic formulas and protein powders were each reported by 36% of respondents as useful tools. Additionally, 27% allowed a more liberal carbohydrate allowance, and 22% allowed greater flexibility in protein targets, to accommodate plant‐based sources that may contain both protein and carbohydrates.

Several practitioners also commented that when working with vegan families, they advised prioritising ‘pure’ fat sources—such as oils—over processed plant‐based fat alternatives (e.g., vegan spreads or creams), which may contain added carbohydrates or protein and could reduce the diet's ketogenic potential.

For patients requiring allergen‐safe ketogenic diets, similar strategies were reported. Use of MCT oils or MCT‐containing products was the most common approach, used by 51% of respondents. Commercial ketogenic formulas were used by 44%, while 19% relied on protein powders. Where needed, 13% allowed a more liberal carbohydrate limit, and 14% adjusted protein targets to support adequacy and tolerability in the context of food allergies or intolerances.

### 3.7 Meal Planning

Translating a ketogenic diet prescription into a practical and sustainable meal plan is a crucial component of dietary implementation. While formats may vary between centres, meal plans are most commonly provided as macronutrient targets for each meal or snack, enabling families to structure their food intake around the prescribed fat, protein, and carbohydrate allowances. Sample meal plans and recipe examples are provided in Tables S5 and S6, demonstrating typical approaches for both CKD and MKD prescriptions. In the MKD examples, the fat content naturally present in high‐protein foods is included in the fat allowance—although this practice may differ between centres.

For MKDs, families may benefit from structured guidance on how to ‘build a meal’ within their macronutrient targets. A common approach involves selecting a protein source, adding a measured portion of vegetables or fruit in line with the carbohydrate prescription, and then incorporating a source of fat to meet the fat target. This framework offers a practical and flexible way for families to assemble meals without requiring full calculation for every ingredient.

Many centers provide choice or exchange lists to further support meal planning. These may include either weighed options or household measurements, depending on local practice. The number of grams of carbohydrate or fat assigned to each ‘choice’ may vary between centers. Examples of such lists are included in Tables S7 and S8.

In addition, some centers provide families with a list of ‘free foods’—items that contain negligible amounts of carbohydrate and may be consumed without being included in the diet prescription. These may include very low‐carbohydrate vegetables (e.g. mushrooms), small amounts of fruit (e.g. lemon), sugar‐free drinks (e.g. squash, cordial, carbonated beverages), and flavorings such as herbs and spices. The criteria for defining free foods differ between centers and should be clearly explained during the education process.

### 3.8 Enteral Nutrition

Enteral nutrition is a common and effective method for delivering KDT, particularly for individuals who are unable to meet their nutritional needs orally. CKD are most frequently used in this context [8], and can be administered via nasogastric, gastrostomy, or jejunostomy tubes. The literature supports both the efficacy and adherence of CKD in enterally fed patients. In a study by Kossoff et al. [44], 59% of children who were exclusively tube‐fed achieved over 90% seizure reduction at 12 months. Similarly, Hosain et al. [45] reported high levels of compliance in this population.

A variety of commercial ketogenic formulas are available to support enteral feeding, including both liquid and powdered preparations (Table S9). The availability, composition, and prescribing processes for these products vary by country. Formulas can be used as a sole source of nutrition or combined with other products to meet the patient's individual energy and nutrient needs.

CKD can also be delivered using blended ketogenic diets (BKDs), which involve liquefying food or drink to a suitable consistency for tube feeding [46, 47]. Recipes for BKDs are typically developed in the same way as for oral diets, with adaptations for texture and viscosity. An example BKD recipe is provided in Table S5.

Although modified ketogenic diets (MKDs) can also be delivered via enteral nutrition—either wholly or in part—their structure and principles are generally more applicable to oral or blended food‐based feeding approaches. When used enterally, MKDs may require additional planning and monitoring to maintain dietary balance and ensure therapeutic efficacy.

#### 3.8.1 Survey Results

Survey findings reflected widespread use of KDT in enterally fed patients. The majority of respondents (91%) reported using a sole‐source complete nutrition formula for patients without allergies receiving enteral ketogenic diets. These formulas provide all necessary macronutrients and micronutrients in a preprepared format, simplifying administration and reducing preparation burden for families and healthcare teams.

However, many centres reported using more flexible or tailored approaches where appropriate. To meet individual nutritional needs and accommodate clinical considerations, 43% of respondents reported combining modular components or blended foods with commercial ketogenic formulas. Additionally, 40% of respondents reported using home‐prepared blended whole foods, and 21% used commercially available blended ketogenic formulas.

For patients with cow's milk protein allergy who require enteral feeding, a broader range of strategies is employed. Modular components were the most commonly used approach, reported by 52% of respondents. Other frequently used options included ketogenic peptide‐based formulas (41%), blended whole foods (40%), plant‐based ketogenic formulas (36%), and commercial blended ketogenic formulas (14%).

These findings highlight the adaptability of enteral ketogenic diet provision across clinical settings, with practices tailored according to product availability, nutritional requirements, allergy status, and local expertise.

### 3.9 Parenteral Nutrition

Parenteral nutrition (PN) may be indicated in clinical situations where enteral feeding is not possible and bowel rest is required – such as gastrointestinal complications or severe illness [48]. Ketogenic parenteral nutrition (KPN) may be required when it is necessary to maintain of ketosis during periods of enteral feeding interruption. KPN is not recommended in preterm infants or malnourished children, due to elevated risk of further malnutrition, metabolic instability, and associated complications [49]. Where KPN is considered, it must be undertaken by a team with specialist expertise in KDT, and only following comprehensive medical and nutritional assessment. The goals of therapy should be clearly defined, and the potential benefits weighed carefully against the associated risks.

Unlike MKDs, which are often structured around flexible macronutrient targets or food‐based prescriptions, KPN requires precise macronutrient calculations. Each macronutrient component must be prescribed to ensure safe intravenous delivery and therapeutic efficacy [49].

Formal guidance on the implementation of KPN has been published elsewhere [49] and teams considering its use should refer to this literature to inform clinical decision‐making and protocol development.

### 3.10 Prescribable Ketogenic Products

Prescribable ketogenic products are commonly used in both CKDs or MKDs to support the diet. These products may be used alongside oral food intake to increase daily fat consumption, modify the type of fat used, and improve intake of protein, vitamins, and minerals [32]. They can be particularly useful in managing complex prescriptions or supporting dietary variety.

In addition to prescribable items, a range of non‐prescribable products—such as low‐carbohydrate baking mixes, cereals, and snacks—may also be suitable for individuals following KDT. The choice of product should be individualised and based on factors including the patient's age, energy requirements, ketogenic ratio, presence of food allergies or intolerances, and any needs for plant‐based or texture‐modified diets.

All prescribable and specialty food products used in KDT should be counted within the diet prescription, and their use should be monitored and moderated to maintain dietary balance and therapeutic efficacy.

#### 3.10.1 Survey Results

According to survey responses, 84% of dietitians offer commercial ketogenic formulas to orally fed patients on KDT, although these are not always used routinely. The availability, prescribing process, and formulation of these products vary between countries and healthcare systems. An outline of commonly used prescribable ketogenic products is provided in Table S9.

Medium‐chain triglyceride (MCT) products are widely used as part of KDT protocols, with 87% of survey respondents reporting their inclusion. Most respondents (78%) reported prescribing or increasing MCTs in millilitre volumes, adjusting according to clinical goals and tolerance.

When asked about the regulation of keto‐specialty food products, 69% of respondents stated they only restrict these items if they are found to negatively affect ketosis or seizure control. A small proportion (7%) reported limiting such products during the first month of treatment, while 6% reported always applying limitations, depending on the individual patient.

### 3.11 Diet Initiation

#### 3.11.1 Classical Ketogenic Diets

Historically, CKDs were initiated in an inpatient setting and preceded by a period of fasting, typically lasting between 12 and 24 h. However, both the literature and current clinical practice no longer support this approach. A large majority of survey respondents (96%) reported that fasting is not required for effective CKD initiation, a position consistent with findings in the literature suggesting that gradual initiation leads to fewer adverse effects without compromising dietary efficacy [50, 51]. As a result, most children and young people can now begin CKD at home, reducing disruption to family life and the need for hospitalisation. Exceptions include infants under 1 year of age [52] and medically complex patients, for whom inpatient monitoring may still be appropriate.

In practice, some clinicians do not aim to immediately reach a specific target ketogenic ratio. Instead, they may adopt a more incremental approach—starting at a lower ratio or a percentage of fat and gradually increasing it over time. This ‘low and slow’ methodology [53] allows for ongoing review of clinical response, ketone levels, and dietary tolerance, facilitating a more personalised and tolerable initiation process.

#### 3.11.2 Survey Results

In clinical practice, the majority of practitioners (79%) report gradually increasing the CKD ratio during the initiation phase, typically progressing from a 1:1, to a 2:1, and then 3:1 ratio. Some practitioners described using smaller incremental steps (e.g. increases of 0.2 to 0.5) based on patient tolerance and response. Adjustments to the ratio are generally made in response to ketone levels and clinical efficacy. A smaller proportion of respondents (15%) initiate CKD by introducing the full dietary prescription one meal at a time. The remaining respondents described using a flexible or combined approach, adapting the method to suit the individual needs and preferences of the patient or family.

The duration of the initiation process was noted to depend largely on the clinical setting. For those initiating the diet in an inpatient setting, more than half of respondents (55%) aim to establish ketosis—defined as ketone levels above 2 mmol/L—within three to 4 days. Some respondents reported using faster or slower initiation protocols tailored to specific clinical scenarios or patient needs.

When initiating CKD via enteral nutrition, most practitioners start with a ketogenic ratio of 1:1 or 2:1 and gradually increase the ratio depending on the degree of ketosis and seizure control achieved. Two in five respondents follow this stepwise approach. An alternative strategy, used by 10% of respondents, involves incrementally substituting the patient's usual enteral formula with a ketogenic formula—typically in steps such as one‐quarter, one‐half, three‐quarters—until the full prescription is in place. A further 38% of respondents reported using a combination of both methods.

Example initiation protocols for both oral and enteral CKD are provided in Tables S10 and S11, respectively.

#### 3.11.3 Modified Ketogenic Diets

There are limited published data detailing specific protocols for the initiation of MKDs. In clinical practice, particularly within the UK, MKDs are typically initiated in an outpatient setting. The introduction of the diet is usually gradual, most commonly over a period of 7 days or less [6]. This approach allows for close monitoring of tolerance and adherence while minimising disruption to family life. Unlike classical ketogenic diets, MKD initiation rarely involves hospital admission or a formal fasting period.

#### 3.11.4 Survey Results

Survey responses revealed a variety of approaches to initiating modified ketogenic diets (MKDs). One‐third of practitioners reported starting with the most liberal macronutrient targets possible and only increasing dietary strictness if needed to improve efficacy. In contrast, 18% of respondents begin with more restrictive macronutrient goals and gradually liberalise the diet as tolerated. For the remaining 40%, the approach depends on individual patient needs, highlighting the importance of flexibility and personalisation in MKD initiation.

The majority of centres (89%) do not use a fasting protocol when initiating MKD. Among the small number who do, fasting typically lasts between 8 and 12 h (7% of centres), with a smaller proportion (6%) reporting fasting durations longer than 18 h.

Regarding the practical method of initiation, 47% of respondents reported beginning the MKD at full calorie targets (where calorie goals are set), with macronutrient goals either introduced immediately or advanced gradually. Similarly, 49% of centres reported introducing the diet one meal at a time.

Time taken to reach the target prescription varied between services. Approximately One‐third (35%) of respondents reported that patients typically reach their full MKD prescription within 3–4 weeks. Others indicated more rapid initiation, with 26% reaching targets in 1–2 weeks, and 17% within less than 1 week.

Examples of MKD initiation approaches are provided in Table S12.

### 3.12 Home Monitoring

#### 3.12.1 Ketones

Monitoring ketone levels at home can be a useful marker to assess whether the dietary prescription is sufficient to induce and maintain therapeutic ketosis. It may also guide any necessary dietary adjustments. However, it is important to note that individuals following MKDs may exhibit lower ketone levels than those on CKDs [54].

Ketones can be measured via urine (detecting acetoacetate) or blood (detecting beta‐hydroxybutyrate, BHB). Urine testing is less invasive and more widely accessible but tends to be less accurate, as results may be affected by hydration status and do not reflect real‐time ketone levels [55]. Nevertheless, urine ketone testing remains a practical option for home monitoring and, when used, should be performed several times per week [9]. Blood ketone testing provides a more accurate reflection of current metabolic state and can be performed both in clinical settings and at home using glucose meters compatible with ketone strips. BHB levels have been shown to correlate more closely with seizure control than urine ketones [56, 57]. Urine ketone levels of 3–4+ (approximately 8–16 mmol/L) typically correspond to blood ketone levels of around 2 mmol/L. Blood ketone concentrations exceeding 3 mmol/L, and in some cases 4 mmol/L, may be most effective for seizure reduction, although the literature remains inconclusive [56, 58, 59]. In infants, routine blood ketone monitoring is recommended to identify potential excess ketosis and mitigate risks in this vulnerable population [52].

##### 3.12.1.1 Survey Results

In clinical practice, the majority of survey respondents (86%) report using blood ketone monitoring when initiating CKDs, with 70% recommending that BHB be tested twice daily. More than half (54%) advise reducing or discontinuing ketone monitoring once levels stabilise, while 23% recommend doing so once treatment goals are achieved. For long‐term monitoring at home, 41% of practitioners rely on urinary ketone testing, supplemented with blood BHB measurements as needed—either at home or during routine clinic visits.

For MKD initiation, over half of respondents (57%) recommend that ketone monitoring should begin with the first MKD meal, whereas 30% advise waiting until the full diet prescription is reached. Just over half (52%) advise testing ketones twice daily during initiation, while One‐third (33%) recommend once‐daily monitoring. Guidance on when to reduce or discontinue ketone testing varies: 53% suggest stopping or reducing once levels are stable, 19% after treatment goals are met, 10% after 3 months on the diet, and 6% within 4 weeks. However, 16% of respondents advise continuing ketone monitoring indefinitely.

In addition to routine monitoring, 89% of respondents recommend checking ketones in the event of symptoms of hyperketosis. Other commonly cited indications include loss of seizure control (88%), signs of illness (66%), and before or after physical activity (14%).

Target ketone ranges vary across practices and are often individualised. However, it is common for clinicians to aim for blood BHB levels between 2 and 6 mmol/L and urinary acetoacetate levels between 8 and 16 mmol/L (personal communication with study authors).

#### 3.12.2 Glucose

Due to the low carbohydrate content of KDT, there is a recognised risk of hypoglycaemia, particularly during the initiation phase or during episodes of illness. While this is a known concern, published reports suggest that hypoglycaemia is rare, with most cases occurring in infants receiving CKD [60, 61, 62, 63]. Nonetheless, careful monitoring during initiation—especially in younger or medically complex children—is recommended to identify and manage any episodes of low blood glucose promptly.

##### 3.12.2.1 Survey Results

Survey responses highlighted that glucose monitoring is common practice when initiating CKD, particularly in inpatient settings. 94% of respondents monitor glucose levels during inpatient initiation, with continued monitoring typically maintained until glucose levels are considered stable (26%). One‐fifth of practitioners continue glucose checks until the full dietary prescription or target ratio is reached, while smaller numbers perform intermittent testing every 2–5 days (6%), weekly (6%), or every one to 2 weeks (8%). There was limited experience reported with continuous glucose monitoring outside of intensive care units.

In outpatient settings, 44% of respondents recommend twice‐daily glucose monitoring at initiation, while 14% recommend daily monitoring and 12% either do not recommend routine glucose checks or advise testing only in response to hypoglycaemia symptoms. Twenty‐two percent of respondents continue outpatient glucose monitoring until levels stabilise, with others advising continuation until ketone levels stabilise (14%) or until the full prescription is reached (14%).

For MKDs, glucose monitoring was reported far less frequently. Only 35% of respondents recommend twice‐daily checks at initiation, 15% suggest once‐daily checks, and 32% do not routinely monitor glucose at all. The remainder tailor their recommendations to the individual patient, often depending on the child's age or presence of symptoms suggestive of hypoglycaemia. Among those who do recommend monitoring, most discontinue checks once glucose levels are stable (26%), while others stop when ketone levels stabilise (7%), when the full prescription is achieved (6%), or after a fixed duration such as 2 weeks (6%).

Definitions of hypoglycaemia varied between centres. The most commonly cited thresholds were < 3.0 mmol/L (23%) or < 2.5 mmol/L (19%), while other respondents used thresholds of < 50 mg/dL (18%) or < 40 mg/dL (11%).

In the event of hypoglycaemia, most practitioners recommend rapid intervention. Three‐quarters (77%) advise a specific volume of sugar‐sweetened beverage, 41% recommend a defined quantity of carbohydrate in grams, 33% use a commercial carbohydrate modular product, and 31% report use of intravenous dextrose. From survey comments, the most commonly suggested oral or enteral intervention was 3–5 g of carbohydrate, often provided as 30–50 mL of fruit juice.

### 3.13 Follow‐up

Optimal clinical management guidelines recommend that children aged over 1 year should receive a clinical review, including laboratory monitoring, at 1 month after initiation, followed by reviews at 3, 6, 9, and 12 months [9]. After the first year, follow‐up visits typically occur every 6 months. Between visits, regular communication with the dietetic team—via telephone or email—is strongly encouraged to monitor progress and address issues promptly. For infants under 1 year of age, more frequent and intensive follow‐up is advised due to their rapid developmental changes and heightened nutritional vulnerability [9].

Each follow‐up appointment should involve a multidisciplinary assessment of clinical and nutritional status. This includes review of serum and urine biochemistry, evaluation of growth parameters (height, weight, BMI, and head circumference in infants), and assessment of adherence to the diet. Nutritional status should be assessed in the context of growth velocity and ideal weight for stature, with consideration of whether the current KDT prescription remains appropriate. Supplementation with vitamins and minerals should also be reviewed and adjusted if necessary.

Nutritional assessments at follow up should include (adapted from optimal clinical management recommendations [9]):

- Height, weight, ideal weight for stature, growth velocity, BMI when appropriate
- Head circumference in infants
- Review appropriateness of KDT prescription
- Review vitamin and mineral supplementation
- Assess compliance to KDT
- Adjust KDT if necessary to improve compliance, seizure control and/or nutritional status

Psychosocial factors identified during pre‐diet preparation—such as family support, cultural considerations, and educational needs—should also be revisited during follow‐up, as these may impact long‐term adherence and outcomes.

If non‐adherence is identified or suspected, a number of strategies can be considered to improve engagement and dietary success. These include practical interventions such as teaching kitchens or cooking demonstrations [19, 64], use of ketogenic applications or calculators [22, 65, 66], trial of alternative dietary regimens such as LGIT [65, 67, 68], and leveraging digital communication tools (e.g. email, WhatsApp) to maintain regular contact and reinforce support between visits [68].

#### 3.13.1 Survey Results

Survey responses indicate broad alignment with optimal clinical management recommendations for follow‐up practices. The majority of practitioners (88%) monitor gastrointestinal health and non‐seizure benefits—including cognition and quality of life (72%)—as part of routine follow‐up. Satiety is also reviewed by two‐thirds of respondents (66%), reflecting an emphasis on the holistic impact of the ketogenic diet beyond seizure control.

In assessing adherence to KDT, ketone levels are the most widely used indicator, with 93% of respondents relying on blood or urinary ketones to gauge dietary compliance. Verbal discussions during clinic appointments or phone calls are also common (91%), with food diaries used by 62% of practitioners. A smaller proportion (12%) make use of formal questionnaires to support their assessment.

Medication weaning decisions vary between centres but are most commonly considered when seizure control is achieved (69%). A third of respondents (33%) indicated that they also consider tapering medication if there is a risk of adverse interactions between antiepileptic drugs and KDT. Timelines for initiating medication reduction vary: 29% consider weaning after 3 months on diet, 13% after 6 months, and 10% as early as 6 weeks, depending on clinical response and practitioner judgement.

#### 3.13.2 Telehealthcare

Telehealthcare has become an increasingly valuable tool in the maintenance of ketogenic diet therapy (KDT), particularly following its expanded use during the COVID‐19 pandemic [69, 70, 71]. While not universally appropriate, it can offer enhanced flexibility and accessibility for families, especially those living at a distance from specialist centres. However, careful consideration should be given to both the advantages and disadvantages of telehealth (outlined in Table S13) when determining its suitability for individual patients and at specific stages of their treatment journey.

There are no changes to the recommended frequency or content of clinical reviews when delivered via telehealth. With appropriate planning, a tailored approach, and support from an experienced multidisciplinary team, KDT can also be successfully initiated remotely for selected patients and families [69, 70, 71]. This flexibility supports continuity of care and broadens access to ketogenic services, while maintaining clinical safety and effectiveness.

##### 3.13.2.1 Survey Results

In clinical practice, telehealthcare is commonly used to support ketogenic diet therapy (KDT), particularly for patients and families who live far from the clinical centre. Over half of survey respondents (62%) reported using telehealth for this reason, while others (23%) indicated it was used as an adjunct for patients requiring additional support. A third of practitioners (32%) use video consultations for all patients, while 39% reported using them specifically for monitoring or review appointments between face‐to‐face visits. Additionally, 37% offer video appointments on an as‐needed basis, and 14% use video for all clinic appointments except for rare instances when in‐person assessment is necessary. 13% conduct one face‐to‐face consultation per year, with all other appointments held remotely.

In addition to video appointments, other forms of remote communication were widely adopted. Telephone contact was the most commonly used (93%), followed by email (80%). Some teams used messaging through electronic medical charts, online personal health records or apps (14%), while others reported providing support via patient support groups (13%), educational videos (13%), newsletters (6%), or messaging services such as WhatsApp (11%).

### 3.14 Fine‐Tuning the Diet Prescription

Fine‐tuning of the ketogenic dietary prescription is an important part of clinical care, allowing practitioners to optimise therapeutic ketosis, improve treatment efficacy, and minimise adverse effects. While often associated with the initial trial or adjustment period, fine‐tuning can be required at any stage during treatment. Adjustments may include changes to macronutrient ratios, energy intake, fluid targets, or supplement use, and are typically informed by clinical response, ketone levels, side effects, and patient or family feedback. Evidence from Selter et al. demonstrated that fine‐tuning led to improved seizure control in 18% of patients already responding to the classical ketogenic diet, with 3% achieving seizure freedom following adjustments [72].

#### 3.14.1 Survey Results

According to our survey, changes to the KDT prescription are most commonly made by the dietitian (69%), or jointly with the wider ketogenic team (20%). In a smaller number of cases (7%), adjustments are led by the medical doctor. The primary driver for fine‐tuning the prescription is seizure control, cited by 95% of respondents for CKD and 85% for MKD. This is followed by ketone levels (70% for CKD, 63% for MKD) and achieving a specific macronutrient ratio or prescription (52% for CKD, 74% for MKD). Survey comments also highlighted the importance of factors such as dietary adherence, acceptability and tolerance, glucose levels, growth, behaviour, and changes in weight. Most practitioners (88%) reported that they consider liberalising the dietary prescription as the patient gets older. This is usually based on clinical diagnosis and progression, the stability of ketosis, duration on the diet, and adherence over time.

##### 3.14.1.1 Fat

Adjustments to fat intake within a KDT prescription are primarily driven by the need to optimise ketosis, manage side effects, and improve seizure control. The majority of survey respondents reported that fat content is modified based on ketone levels (90% for CKD, 84% for MKD), gastrointestinal side effects (90% for CKD, 84% for MKD), and seizure control (81% for CKD, 84% for MKD). Other influencing factors include the presence of hyperlipidaemia (reported by 77% of CKD respondents and 72% of MKD), weight change (59% CKD, 56% MKD), and the need to improve dietary adherence (57% CKD, 54% MKD). To a lesser extent, changes in blood glucose levels (23% CKD, 25% MKD) and linear growth (21% CKD, 25% MKD) may also guide fat prescription adjustments.

MCTs are commonly used to support fine‐tuning of KDT, as their metabolism yields more ketones per gram than LCTs, owing to their rapid absorption and hepatic conversion. 95% of practitioners reported using MCT as part of this process, most frequently when ketone levels are suboptimal or inconsistent (96%), or when further restriction of carbohydrate is poorly tolerated (48%). Based on survey comments, MCT is typically prescribed up to 30%–50% of total calories in CKD and up to 60% in MKD, depending on individual tolerance and clinical judgement. Introduction is often gradual due to the risk of gastrointestinal side effects, including abdominal discomfort, loose stools, nausea, and vomiting [73].

##### 3.14.1.2 Carbohydrate

Carbohydrate intake is often adjusted during KDT to improve efficacy, metabolic control, and diet tolerability. According to our survey, the most common reason for modifying carbohydrate prescription is to address suboptimal ketone levels, cited by 89% of respondents for CKD and 82% for MKD. Adjustments may also be made when seizure control or other treatment goals are not being met (69% CKD, 78% MKD), or to support better blood glucose regulation (82% CKD, 69% MKD). Improving adherence to the diet is another key driver (72% CKD, 62% MKD), as is the need to mitigate adverse effects such as gastrointestinal discomfort or poor appetite (61% CKD, 59% MKD). In some cases, carbohydrate is adjusted in response to weight changes (38% CKD, 56% MKD) or concerns about linear growth (23% CKD, 25% MKD).

When fine‐tuning carbohydrate intake, it is important to account for nonnutritive sources that may influence ketone production. Ingredients such as sugar alcohols—commonly found in low‐carbohydrate or ‘keto’ food products, drinks, and some liquid medications—can impact ketosis and should be included in dietary assessments.

##### 3.14.1.3 Protein

Protein prescriptions in KDT are adjusted based on a range of clinical indicators and patient needs. Survey results show that the most common reason for modifying protein intake is to support linear growth, cited by 73% of respondents for CKD and 62% for MKD. Suboptimal ketone levels are also a factor (50% CKD, 54% MKD), as are laboratory markers such as low serum urea or abnormal amino acid profiles (59% CKD, 49% MKD). Adjustments may also be made to improve dietary adherence (51% CKD, 49% MKD), respond to lack of efficacy in terms of seizure control or other clinical goals (33% CKD, 44% MKD), or address weight changes (54% CKD, 42% MKD). Less commonly, adverse effects (24% CKD, 27% MKD) and blood glucose levels (18% CKD, 23% MKD) influence the decision to amend protein targets.

In children receiving CKD via enteral nutrition, the choice of protein source may also be considered. For example, using whey‐based formulas instead of casein‐based ones can be beneficial in managing reflux symptoms [74].

### 3.15 Adverse Effects

Potential adverse effects associated with KDT are detailed in published optimal clinical management recommendations [9]. Gastrointestinal symptoms are the most frequently reported side effects during the early stages of treatment and are typically short‐lived or manageable through dietary modifications and, if necessary, medical intervention. In the short term, individuals on CKD may also experience alterations in micronutrient status or episodes of hypoglycaemia [36, 63]. Over the longer term, more serious complications can arise, including hyperlipidaemia, renal calculi, reduced bone mineral density, hypercalciuria, growth concerns, cardiac abnormalities, and in rare cases, pancreatitis [9, 75]. These adverse effects require careful monitoring and multidisciplinary management. While medical oversight is essential, adjustments to the ketogenic diet can play a role in mitigating some of these risks. A range of potential dietary strategies for addressing persistent symptoms and adverse effects is summarised in Table 5.

**Table 5:** Dietary adjustments for individuals on ketogenic diet therapy with specific symptoms/adverse effects.

Symptom	Considerations	Strategies (% of survey respondents who selected each strategy)
High ketone levels and/or acidosis	Is calorie intake sufficient/weight gain appropriate?	Increase carbohydrate intake (80%)
	Any recent weight loss?	Reduce fat intake (73%)
	Any missed meals or feeds?	Increase fluid intake (54%)
	Any delay in meals or feeds?	Start bicarbonate (38%)
	Any recent changes in ingredient brands used?	
	Any recent changes in medication?	
	Any signs of illness?	
	Evaluate fluid intake/dehydration	
	Evaluate which fat sources are being used	
Low ketones	Is calorie intake too high/is there rapid weight gain?	n/a (not asked in survey)
	Is protein intake excessive?	
	Check adherence to diet prescription	
	Check if there are any recent change in products or ingredient brands	
	Any signs of illness?	
	Any recent changes in medication?	
	Any changes in family situation/routine/travel?	
	Any pattern as to which time of day ketones are lower/higher?	
	Any change to bowel habits? Constipation can slow digestion and impact ketosis.	
Recurrent hypoglycemia	Check adherence to diet prescription	Increase carbohydrate intake (86%)
	Check for any changes in activity levels	Increase daily calories (58%)
	Check for patterns of hypoglycemia	Decrease fat intake (28%)
	Evaluate frequency of meals/snacks	Provide a specified amount of sugar‐sweetened beverage or commercial carbohydrate modular component every time hypoglycemia occurs (20%)
	Check for level of ketosis	Increase protein intake (18%)
	Check energy intake and growth	
	Evaluate feed dilution, where applicable	
Diarrhea	Any new medications recently started, in particular antibiotics? If so, could this be linked?	Reduce MCT (86%)
	Any new foods/ingredients/fat sources introduced recently?	Supplementation with probiotics (60%)
	Consider feeding rate (enteral feeders)	Increase fluid intake (57%)
	Check adherence to diet prescription	Reduce fat intake (41%)
	Check food portion or feed volume	Lower diet ratio/liberalize diet prescription (39%)
	Check for history of constipation, as could be overflow diarrhea	Decrease fiber from food (33%)
	Evaluate signs of steatorrhea	Supplementation with modular fiber (29%)
	Consider bacterial overgrowth as a potential differential	Increase fiber from food (26%)
Vomiting	Check for excess ketosis or hypoglycemia	Lower diet ratio/liberalize diet prescription (76%)
	Any new foods/ingredients introduced recently?	Reduce fat intake (56%).
	Consider feeding amount and rate (enteral feeders)	Oral rehydration solution mixed with water (44%)
	Check adherence to diet prescription	Increase fluid intake (34%)
	Check food portion or feed volume	Provide electrolyte solution (33%)
	Consider reflux and/or constipation	Suggest periods of fasting (18%)
		Other (27%), for example, consider constipation or reflux, look at timings and volume of meals, trial a peptide formula if applicable.
Constipation	Check fluid intake	Increase fluid intake (96%)
	Check fiber intake	Advise on fiber‐rich food sources (80%)
	Evaluate usual bowel habits	Add MCT (58%)
	Consider recommending smaller meals	Supplementation with modular fiber (49%)
	Chronic constipation should be discussed with the medical team to consider treatment options	Supplementation with probiotics (40%)
		Supplementation with magnesium citrate (23%)
		Offer prune juice (8%) [consider amount required without impacting ketone/glucose levels or seizure control]
Reflux	Evaluate pattern of symptoms	Sit upright when eating/feeding (91%)
	Check food portion or feed volume	Smaller meals/feeds (91%)
	Consider enteral feeding pattern or rate	Reduce fat intake (53%)
	Consider types of fat	Lower diet ratio/liberalize diet prescription (47%)
	Consider constipation	Consider introduction of a thickener (34%)
	Is reflux optimally medically managed, if applicable?	Ginger tea (11%)
		Decrease calories (9%)
Hyperlipidemia	Were laboratory measures taken in a fasted state?	Advise on alternative fat sources (94%)
	Dietary assessment to identify current fat sources	Add MCT (60%)
	Check carnitine levels	Lower diet ratio or liberalize diet prescription (46%)
	Consider that, in many cases, lipid values will stabilize or normalize without intervention within approximately 12 months	Other (19%), for example, encourage food sources of soluble fiber, add plant stanols
	In cases of persistent hyperlipidemia, referral to the metabolic team may be required for genetic testing and/or further investigation	Stop KDT (6%)
Hypercalcemia or hypercalciuria	Assess fluid intake (increased hydration is recommended for individuals with hypercalcemia, with calcitonin as the next therapeutic option [75]	Increase fluid intake (63% re. hypercalcemia/70% re. hypercalciuria)
	Assess calcium intake	Refer to renal specialty (50%/51%)
	Review level of ketosis	Lower supplement dose (49%/40%)
	Consider citrate supplementation	Add a citrate (38%/48%)
	Consider contributing factors, including carbonic anhydrase inhibitors	Lower diet ratio or liberalize diet prescription (37%/40%)
	Consider referral for medical support, if persistent	

### 3.16 Management of Illness

During episodes of intercurrent illness, dietary adjustment may be necessary to maintain ketosis and reduce the risk of seizure recurrence. In such cases, a ketogenic ‘milkshake’ tailored to the individual's prescription can be used as a temporary meal replacement, and may be diluted if poorly tolerated [76]. Close monitoring of both blood glucose and ketone levels is advised, as the risk of hyperketosis or hypoglycaemia is heightened during illness, particularly if dietary intake is reduced [76].

As with any unwell child, medical advice should be sought if there are concerns or the illness persists, to ensure appropriate treatment is provided. Ideally, medications—including antibiotics—should be in low‐carbohydrate formulations. However, if no suitable alternative is available, the medication should not be withheld; treating the underlying illness must take priority. If long‐term treatment with carbohydrate‐containing medication is required and ketosis is affected, the dietary prescription may need to be temporarily adjusted.

Maintaining hydration is also a priority. Sugar‐free fluids should be encouraged, and if needed, rehydration solutions can be used in diluted form to avoid exceeding the individual's carbohydrate allowance. These may help prevent hyperketosis and/or acidosis, particularly in children who are unable to tolerate full meals. If vomiting or diarrhoea occurs, a temporary reduction in fat intake—particularly MCT—may be necessary. This can be achieved by adjusting the diet prescription or offering half‐sized meals or feeds.

If the child is nil by mouth for over 12 h, such as in preparation for surgery, it is advisable to check whether the anaesthetic team has access to a ketogenic protocol [77]. During this time, ketone and blood glucose levels should be monitored more frequently (every 4 h is recommended) to reduce the risk of hypoglycaemia or excessive ketosis. The diet should be reintroduced as soon as the child is able to tolerate food again. If intravenous fluids are required, non‐dextrose‐containing solutions should be used, unless dextrose is necessary to manage acute hypoglycaemia or hyperketosis [76]. Studies have shown that children on KDT undergoing general anaesthesia with carbohydrate‐free intravenous fluids maintained stable glucose levels; however, some experienced metabolic acidosis [77]. As such, serum pH or bicarbonate levels should be monitored during prolonged procedures. If oral or enteral feeding must be withheld for more than 48 h, PN may be considered [49, 78].

#### 3.16.1 Survey Results

In the event of illness, survey respondents reported several common strategies to support patients while maintaining dietary goals. The overwhelming majority (93%) prioritise maintaining adequate fluid intake during illness. Many also reported adapting meal formats to improve tolerability: 78% offer smaller meals or feeds, and 57% recommend the use of oral rehydration solutions to support hydration and electrolyte balance. Just over half of respondents (51%) use ketogenic meal replacement shakes, tailored to the individual's diet prescription, as a temporary alternative when full meals are not tolerated. A smaller proportion (21%) reported offering meals or feeds without added fats, that is, not fully within the ketogenic diet prescription, to help manage intake during periods of gastrointestinal upset or reduced appetite.

### 3.17 Diet Discontinuation

#### 3.17.1 When to Consider Discontinuing KDT?

The decision to discontinue KDT should be made on on an individual basis, involving shared discussion between the child (where appropriate), their parents or carers, the dietitian, and the neurologist. As a medical therapy, KDT carries the risk of adverse effects and should not be continued if treatment goals are not being achieved. Evidence suggests that 75% of children who will respond to KDT do so within the first 14 days [79], though some may take longer (up to 2 months or more) to show benefit. Optimal clinical management recommendations advise discontinuing the diet after approximately 3 months if there has been no reduction in seizures or other therapeutic benefits [9]. In such cases, particularly for patients on MKD, a transition to CKD may be considered to assess whether a stricter regimen may yield improvement [80].

For individuals who do respond well—typically defined as achieving a seizure reduction of 50% or more—discontinuation is often considered after around 2 years of treatment [9]. However, there is no defined maximum duration for KDT [9] and long‐term use has been documented for up to 20 years in individuals experiencing significant seizure control with minimal side effects [81, 82]. In certain cases, longer treatment durations may be appropriate. For example, children with epileptiform discharges on EEG, focal abnormalities on MRI, or a diagnosis of tuberous sclerosis may benefit from extended therapy due to a higher risk of seizure recurrence [83]. Additionally, for individuals with GLUT1 deficiency syndrome (GLUT1‐DS), it is recommended that the diet be continued at least until puberty [9] or or into adulthood [84]. In those with pyruvate dehydrogenase deficiency (PDHD), extended use beyond 2 years may also be appropriate depending on response and tolerability [9].

#### 3.17.2 How to Discontinue KDT?

Discontinuation of KDT should be carefully planned and individualised based on patient response, clinical condition, and the duration of therapy. A retrospective review suggested that a gradual weaning period of 4–6 weeks is both feasible and well tolerated in most cases [85]. For infants on CKD, the ratio should be slowly reduced [52, 86] until ketones are no longer present. A complete wean within 2 weeks may be considered for infants who have derived no clinical benefit from the diet [52].

Once ketone levels fall—typically defined as < 1 mmol/L (< 20 mg/dL) or < 0.5 mmol/L (< 5 mg/dL)—the transition back to the child's usual diet can be accelerated [52, 83, 86]. If seizure control deteriorates during discontinuation, many patients can regain seizure stability through dietary or antiseizure medication adjustments [83, 85]. In such cases, practitioners may opt to pause the weaning process for 3–7 days, reassess clinical response, and then resume at a slower pace. Alternatively, returning temporarily to the previous, better‐tolerated dietary prescription before resuming the wean may be helpful. In all cases, medical input is recommended if seizure frequency increases.

According to optimal clinical management recommendations, micronutrient supplementation should be continued until KDT is fully discontinued [9].

##### 3.17.2.1 Survey Results

Survey responses indicate that the length of time taken to discontinue ketogenic diet therapy (KDT) is highly individualised and influenced by several patient‐specific factors. The most common determinant was the clinical response to the diet, including seizure control and achievement of other therapeutic goals, cited by 91% of respondents. Seventy‐five percent noted that the total duration on KDT influenced the weaning timeline, while 67% reported that patient or family preference played a role. Additional factors included any adverse effects experienced while on the diet (65%), level of ketosis achieved (55%), type of ketogenic diet used (43%), mode of feeding (33%), and the patient's age (with 16% taking longer to wean younger patients, although 5% reported the opposite). A smaller proportion (14%) also considered the epilepsy syndrome when planning discontinuation.

For patients on CKDs, 40% of respondents reported weaning by reducing the ketogenic ratio by 0.5 at each step (e.g., 4:1 to 3.5:1 to 3:1), whereas 34% preferred to reduce the ratio by 1.0 per step. The remaining 26% used alternative or fully individualised protocols. For patients who had only been on the diet for a short time, weaning typically occurred over 1–4 weeks, while longer or more gradual reductions were reported for those on CKD for 2 years or more (Table 6). Example weaning protocols for both oral and enteral CKD are outlined in Tables S14 and S15.

**Table 6:** Length of time to wean off classical ketogenic diets: survey responses.

Patients following CKD for at least 3 months	Agreement rate (% survey respondents)	Patients following CKD for at least 2 years	Agreement rate (% survey respondents)
1–2 weeks	33%	1–2 weeks	3%
3–4 weeks	25%	3–4 weeks	16%
Very individualized	27%	Very Individualized	45%
Less than 1 week	10%	1–2 months	22%
1–4 months	5%	4–6 months	5%

A variety of approaches were described for discontinuing MKDs. Over half of survey respondents (51%) reported that they increase carbohydrate intake and decrease fat intake simultaneously in a stepwise fashion. Another 37% increased carbohydrate first, and only 6% decreased fat first. One common approach when using food choice or exchange lists was to reduce fat choices by one or two per day and increase carbohydrate choices by 1 g per meal or snack every 1–7 days. For patients on MKD for a short period with no observed benefit, changes may be made more quickly (e.g., every 1–2 days), or MKD meals and snacks may be gradually replaced with standard meals over a number of days. Table 7 summarises the reported duration for weaning off MKDs, which, similar to CKDs, tended to be longer for patients who had followed the diet for at least 2 years, and was frequently individualised.

**Table 7:** Length of time to wean off modified ketogenic diets: survey responses.

Individuals following an MKD for approximately 3 months with no/minimal response	Agreement rate (% survey respondents)	Individuals following an MKD for at least 2 years with good response	Agreement rate (% survey respondents)
< 1 week	20%	< 1 week	2%
1–2 weeks	38%	1–2 weeks	5%
3–4 weeks	16%	3–4 weeks	23%
1–2 months	5%	1–2 months	18%
3–4 months	0%	3–4 months	5%
Very individualized	22%	4–6 months	2%
		> 6 months	3%
		Very individualized	41%

Table S16 outlines worked examples of how to discontinue MKDs, presenting two distinct approaches: one that gradually adjusts the daily carbohydrate and fat intake, and another that replaces individual MKD meals with typical or higher‐carbohydrate meals over time. For both methods, two scenarios are illustrated [1]: for patients who have been on MKD for a short time or have shown no clinical response, and [2] for those who have followed the diet longer and/or demonstrated a good response in seizure control.

During the weaning process, over half of survey respondents (64%) continue to monitor ketone levels, while 23% do not. The remainder either leave the decision to families or monitor only until ketosis is no longer present. Regarding vitamin and mineral supplementation, 87% of respondents reported stopping supplementation at the end of the weaning process, whereas 5% only stopped once ketosis was lost. It is important to note that some form of supplementation may still be required for reasons unrelated to the ketogenic diet itself, such as concurrent medications or baseline nutritional status, and ongoing monitoring should be determined by local clinical practice, the healthcare system, and the responsibilities of the individual service or centre.

#### 3.17.3 Dietary Advice After Discontinuing KDT

Following a successful period on KDT, some families may be hesitant to reintroduce carbohydrates (particularly processed or refined sources) due to concerns about seizure recurrence or other negative effects. This is an important consideration when supporting families in transitioning back to a more typical dietary pattern.

Survey findings reflect a wide range of professional approaches. One‐third of respondents recommend avoiding processed and refined sugars altogether when returning to a standard diet. Others take a more gradual approach: 21% advise slowly reintroducing simple carbohydrates, while 14% support gradual reintroduction of processed and refined sugars specifically. A smaller proportion (9%) advise avoiding simple carbohydrates entirely. Notably, 14% of respondents do not provide specific guidance on carbohydrate reintroduction, underscoring the variation in practice and the importance of tailoring advice to individual patient and family needs.

#### 3.17.4 Follow‐up Post Diet Discontinuation

Post‐diet discontinuation follow‐up practices vary between services and are often influenced by local resources and patient needs. According to survey data, 34% of respondents discharge orally fed patients from their service once they are fully weaned off the KDT. Others continue to provide follow‐up support for a period after discontinuation: 18% discharge patients more than 4 weeks after weaning is complete, 14% after two to 4 weeks, and 13% within one to 2 weeks.

Decisions regarding continued follow‐up are typically guided by individual clinical considerations, such as concerns about nutritional status or growth. In many cases, ongoing support may transition to general or community dietetic services, where available, to ensure continuity of care. This highlights the need for effective handover and clear communication between specialist ketogenic teams and broader healthcare services.

## 4 Results: Dietetic Practice Core Recommendations

Table 8 outlines the core recommendations for CKD and MKDs, based on published evidence or survey consensus (≥ 75% agreement rate).

**Table 8:** Core recommendations for classical and modified ketogenic diets.

Classical ketogenic diets	Source	Modified ketogenic diets	Source
**Definition of ketogenic diet type**			
Any ketogenic diet that is based on a ratio of grams of fat to grams of protein and carbohydrate combined, usually in a ratio range of 2:1 to 4:1.	L1	MKDs encompass all ketogenic diets that do not fit the definition of a CKD or a Medium Chain Triglyceride (MCT) KD.	L1
		MKDs are most commonly defined as ketogenic diets that have a carbohydrate limit and a fat target, whereas protein is allowed freely or has a more general age‐specific target.	S; L1
**Patient selection (dietary factors)**			
CKD is preferred over MKDs in patients who are tube fed and for children aged under 2 years.	S	MKDs are preferred over CKD in the case of patient/family preference, for orally fed patients, and those aged over 12 years.	S
		MKDs may be preferred over CKD for vegetarian and vegan diets, but this is patient dependent.	S
**Pre‐diet preparation**			
Patients may benefit from pre‐diet dietary adjustments before starting KDT, for example, by reducing simple sugars, trying out high‐fat foods or ketogenic recipes.			S
At least one education session is required, following agreement from the patient/family and the multidisciplinary healthcare team that KDT will be started.			S
At a minimum, education session should include the following topics:			
Importance of adherence to the diet			
Potential adverse effects			
Follow up expectations			
Identifying carbohydrates, protein, and fat sources			
Weighing/measuring of foods and/or formula			
Importance of hydration			
Identify other sources of carbohydrate, such as medications			
Supplements			
Time commitment needed			
**Diet prescription (energy, fat, protein, carbohydrate, fluid)**			
Energy requirements are based on standardized energy equations, current feeding regimen, and growth trends.	S; L1	Although calorie prescriptions are typically not given for MKDs, if energy requirements are calculated, they are based on standardized energy equations, current feeding regimen and growth trends.	S; L1
Protein requirements should meet the dietary reference intake for age and weight, as a minimum and protein intake should be prioritized over carbohydrate. Sometimes, intakes below the dietary reference intake can be used in the short term.	S; L1	Protein is not routinely prescribed in MKDs, but care should be taken to ensure minimum daily requirements are met.	S; L1
		Fat targets are most commonly 60‐80% total energy. Alternatively, the fat target may be set as any remaining calories after calculating carbohydrate and protein goals.	S; L1
		When setting carbohydrate goals for MKDs, the amount is commonly set based on the age of the patient. Carbohydrate allowances mostly range between 10 – 20 grams per day (total or net carbohydrate can be used).	S; L1
		Fat and carbohydrate should be spread evenly throughout the day.	S; L1
For modified texture diets, low‐carbohydrate options, such as gum‐based thickeners, may be used.			S
Fluid requirements should not be restricted.			S; L1
The ketogenic dietitian is typically responsible for advising on micronutrient supplementation, commonly a complete multivitamin and mineral supplement, plus additional supplements as required, such as carnitine (if low), calcium, and vitamin D.			S; L1
**Enteral nutrition**			
Nutritionally complete ketogenic enteral formulas should be used for those without allergies, including infants that are tube fed.	S; L1		
**Prescribable ketogenic products**			
MCT oil and MCT‐based products can be used as part of CKD and MKDs and the amount should be increased incrementally.			S
For infants without allergies, an age‐appropriate sole‐source complete nutrition formula can be used.	S	Commercial ketogenic formulas can be offered to orally fed patients on MKDs.	S
For infants with cow's milk protein allergy, modular components can be used.	S	Consider limiting keto specialty food products if they seem to negatively impact ketosis or seizure control.	S
**Diet initiation**			
A gradual initiation without an initial fasting period helps reduce the risk of adverse effects and does not impact diet efficacy.	S; L1		
The KD ratio can be gradually increased, for example, from 1:1, to 2:1, to 3:1, depending on individual tolerance and response.	S; L1	MKDs tend to be started at full calories and advanced either by adding in one MKD meal at a time at target macronutrient goals, or by advancing per macronutrient goal, with the aim of reaching the target prescription typically between 1‐4 weeks.	S; L1
Glucose should be monitored when the diet is started as an inpatient.	S		
Glucose *may* be monitored initially when starting the diet as an outpatient.	S		
Ketones should be tested twice daily initially, and testing can be discontinued once ketones are stable.	S; L1	Ketones should be monitored (once or twice daily) during the initiation phase, until ketone levels and the diet prescription are as stable as possible.	S; L1
Additional ketone testing is recommended in the event of signs of hyperketosis, illness or increase in seizures.	S; L1		
**Follow‐up, monitoring and fine‐tuning**			
Offer in‐person clinic visits, or virtual clinic visits (where appropriate) at a minimum of 1, 3, 6 and 12 months post diet start, then 6‐monthly.			L1
Provide additional follow‐up phone calls and/or emails as needed.			L1
At follow‐up appointments, anthropometrics, the nutritional adequacy of the diet prescription, diet adherence and any non‐seizure benefits from KDT should be reviewed.			L1
Adherence is measured by monitoring ketone levels and through discussions with patients/families.			S; L1
Additional ketone checks may be done in the case of symptomatic hyperketosis, loss of seizure efficacy or during illness.			S
The CKD should be fine‐tuned based on individual seizure response and tolerance.	L1		
Consider adjusting the KD ratio, depending on diet efficacy, ketone levels, glucose levels, blood lipid profile and gastro‐intestinal side effects.	S		
Consider use of MCT to promote higher levels of ketosis, if other changes to the diet prescription do not result in seizure improvement or if further restrictions in carbohydrate intake is not tolerated or possible.			S
The diet prescription may be liberalized as patients get older, if needed.			S
**Intercurrent illness**			
In case of constipation, increasing fluid intake and advise on fiber‐rich food sources are considered.			S; L1
For the treatment of acute hypoglycemia, a specified amount of a sugar‐sweetened beverage, such as juice, or a specific amount of carbohydrate (grams) is recommended.			S
In case of recurrent hypoglycemia, increasing carbohydrate intake in diet prescription is considered by dietitians is recommended.			S
In case of illness, prioritize fluid intake, in some cases with an oral rehydration solution, and temporarily offer snack portions in place of full calorie meals.			S
In case of hyperlipidemia, changing dietary fat sources is considered.			S; L1
Meal replacements with ketogenic formula may be helpful in the interim.	L1		
**Diet discontinuation**			
There is no maximum amount of time that KDT can be followed but, if effective, diet discontinuation can be considered after approximately 2 years.			S; L2
If on diet for a short period of time (< 3 months) with no/minimal response, KDT is most commonly weaned over 1‐4 weeks.			S
If on diet for a longer period of time (> 2 years) with good response, the length of time over which KDT is discontinued is most commonly individualized to each patient, but is generally longer than those on diet for short periods of time.			S
The KD ratio may be gradually reduced, for example, by 0.5 or 1 at each step; smaller steps may be used for individuals with good seizure control.	S; L1	When discontinuing MKDs, the most common method is to increase carbohydrate *and* decrease fat in a stepwise fashion. For example, increase carbohydrate by 1 g per meal/snack and reduce fat by one or two ‘choices’ every 1‐7 days	S
Continue monitoring ketones whilst discontinuing KDT, but once ketone levels are minimal, the speed of transition back to the patient's usual diet can be increased.			S
Vitamin/mineral supplementation should be continued until the end of the diet discontinuation process.			S
Patients may be advised to avoid processed and refined sugars when they return to their usual diet, in in line with general healthy eating guidelines, or to gradually re‐introduce simple carbohydrates.			S
**S = survey consensus (≥ 75% agreement); L1 = published consensus recommendations or international guidelines; L2 = published surveys, systematic reviews, meta‐analyses, or randomised controlled trials**			

## 5 Discussion

These recommendations represent the first international best practice guidance for the dietetic management of children and young people with epilepsy following CKDs and MKDs. They are grounded in a combination of published evidence and the most commonly reported dietetic practices, while also acknowledging and valuing the diversity of approaches used across different countries and healthcare systems.

The document is intended as a practical resource for ketogenic dietitians and nutrition professionals involved in the care of children and adolescents receiving medically advised CKDs or MKDs for epilepsy management. While the recommendations seek to support greater consistency and quality in dietetic care worldwide, they are not prescriptive or mandatory, and clinical judgement remains essential. Local policy, patient preference, and resource availability should all be considered when applying these recommendations in practice.

Evidence to support dietetic management of KDT remains limited, particularly for MKDs. The recommendations therefore reflect the best available evidence at the time of writing, alongside expert opinion and international survey data. As with all survey‐based research, certain limitations must be acknowledged. These include the potential for sampling and response bias, and a relatively restricted set of response options. While we achieved a substantial number of respondents globally, the survey was designed to capture individual practice, which may not always represent centre‐wide protocols. In cases where multiple dietitians from a single centre contributed, there is a possibility of overrepresentation of certain practices. However, this also reflects the reality that dietetic practice can vary between professionals even within the same institution.

## 6 Conclusion

Despite the limitations discussed, these recommendations are highly relevant to practicing dietitians and offer a valuable resource for supporting children and young people on CKDs and MKDs. By promoting consistency in practice, they aim to enhance patient and family experience, support adherence to dietary therapy, and potentially improve clinical outcomes. Future research should prioritise the perspectives of service users and seek to evaluate the clinical impact of differing dietetic approaches, to further strengthen the evidence base and optimise care.

## Author Contributions

Conceptualization: N.E.S. Methodology: N.E.S., V.J.W., L.C.L.N., M.G. Survey creation, M.J., N.E.S., V.J.W., L.C.L.N., M.G. Organization of literature review group: L.C.L.N., M.G. Writing – extracting information from literature review and survey. All authors, not including MKD best practice recommendations advisory group. Writing – original draft preparation: N.E.S., V.J.W., L.C.L.N., M.G., R.B., C.T‐S. Writing – review and editing, all authors, including MKD best practice recommendations advisory group. Funding acquisition: N.E.S. All authors have read and agreed to the published version of the manuscript.

## Best Practice Recommendations Advisory Group, on behalf of the Ketogenic Dietitians Research Network

Christine Au Yeung, Oakland Medical Center, Oakland, California, USA. Tessa Bollard, Sydney Children's Hospital, Sydney, New South Wales, Australia. Ramona De Amicis, University of Milan, Milan, Italy. Diana Lehner‐Gulotta, UVA Health Charlottesville, Virginia, USA. Nicole Mills, Cambridge University Hospitals, Cambridge, UK. Candy Richardson, Duke University Hospital‐The Children's Health Center Durham, North Carolina, USA. Christi Sports, Phoenix Children's, Phoenix, Arizona, USA. Lisa Vanatta, Phoenix Children's, Phoenix, Arizona, USA. Kristel Van de Kerckhove, UZ Leuven, Leuven, Belgium. Rocio Viollaz, Hospital de niños Sor M. Ludovica, La Plata, Argentina. Sze Man Wong, Royal Manchester Children's Hospital, Manchester, UK.

## Conflicts of Interest

The authors declare no conflicts of interest. The funders had no role in the design of the study; in the collection, analyses, or interpretation of data; in the writing of the manuscript; or in the decision to publish the results.

## Supporting information


**Table S1:** Search terms. **Table S2**: Components of modified ketogenic diets (% agreement rate from survey respondents). **Table S3**: Carbohydrate allowances in initial modified ketogenic diet prescription for different age ranges (% agreement rate from survey respondents). **Table S4**: List of commonly used ingredients and suggested alternatives for individuals with special dietary requirements. **Table S5:** Example classical ketogenic diet daily meal plan. **Table S6**: Example modified ketogenic diet daily meal plan. **Table S7:** 5g fat (long‐chain triglyceride) choices for Modified ketogenic diets. **Table S8:** 1g carbohydrate choices for Modified ketogenic diets. **Table S9**: List of ketogenic prescribable products. **Table S10**: Example initiation schedules for an oral classical 3:1 ketogenic diet. **Table S11:** Example 3‐week initiation schedule for an enteral classical 3:1 ketogenic diet. **Table S12:** Example initiation schedule for a modified ketogenic diet. **Table S13:** Advantages and disadvantages of the use of telemedicine for ketogenic diet therapy. **Table S14:** Worked examples of how to discontinue a classical ketogenic diet. **Table S15:** Worked examples of how to discontinue an enteral classical ketogenic diet. **Table S16:** Worked examples of how to discontinue a modified ketogenic diet.

## Data Availability

The data that supports the findings of this study are available in the supporting material of this article. The original contributions presented in this study are included in the supporting material. Further inquiries can be directed to the corresponding author(s).
