# Supplementary material for: Classical and Modified Ketogenic Diets for Children and Young People With Drug‐Resistant Epilepsy: A Reflection of International Dietetic Practice and Best Practice Recommendations for Dietitians
Source: J Hum Nutr Diet. 2025 Oct 12;38(5):e70129. doi: 10.1111/jhn.70129 (PMC12516026; doi:10.1111/jhn.70129)
Supplement: Supplementary file 1 — Table S1: Search terms. Table S2: Components of modified ketogenic diets (% agreement rate from survey respondents). Table S3: Carbohydrate allowances in initial modified ketogenic diet prescription for different age ranges (% agreement rate from survey respondents). Table S4: List of commonly used ingredients and suggested alternatives for individuals with special dietary requirements. Table S5: Example classical ketogenic diet daily meal plan. Table S6: Example modified ketogenic diet daily meal plan. Table S7: 5g fat (long‐chain triglyceride) choices for Modified ketogenic diets. Table S8: 1g carbohydrate choices for Modified ketogenic diets. Table S9: List of ketogenic prescribable products. Table S10: Example initiation schedules for an oral classical 3:1 ketogenic diet. Table S11: Example 3‐week initiation schedule for an enteral classical 3:1 ketogenic diet. Table S12: Example initiation schedule for a modified ketogenic diet. Table S13: Advantages and disadvantages of the use of telemedicine for ketogenic diet therapy. Table S14: Worked examples of how to discontinue a classical ketogenic diet. Table S15: Worked examples of how to discontinue an enteral classical ketogenic diet. Table S16: Worked examples of how to discontinue a modified ketogenic diet. [file JHN-38-e70129-s001.docx]

**Table S1:** Search terms

| **Search topic** | **Search terms** | **Date of last search** |
| --- | --- | --- |
| Patient selection | (child* OR infant) AND (ketogenic OR "modified atkins") AND Epilepsy[MeSH Terms] AND (patient selection [MeSH Terms] OR Nutrition Assessment[MeSH Terms] OR contraindications[MeSH terms] OR "indication*") | 14/02/2024 |
| Patient selection (diet types) | (child* OR infant) AND (ketogenic OR "modified atkins") AND Epilepsy[MeSH Terms] AND (diet selection [MeSH Terms] OR Nutrition Assessment[MeSH Terms] OR contraindications[MeSH terms] OR "indication*") | 22/02/2024 |
| Diet prescription | (child* OR infant) AND (ketogenic OR "modified atkins") AND Epilepsy[MeSH Terms] AND ("diet prescription" OR "grams" OR "percentage energy" OR macronutrient* OR micronutrient* OR "ratio*") | 12/02/2024 |
| Mode of feeding | (“child*” OR “infant”) AND (“ketogenic” OR "modified atkins") AND Epilepsy[MeSH Terms] AND (“enteral” OR “tube” OR “blended” or “gastrostomy”) | 24/01/2024 |
| Adolescents and ketogenic diets | (ketogenic OR "modified atkins") AND Epilepsy[MeSH Terms] AND (Infant[MeSH Terms] OR Child[MeSH Terms] OR Adolescent[MeSH Terms]) AND (social OR adapt*) | 22/02/2024 |
| Differences in practice with patients of different age group | (ketogenic OR "modified atkins") AND Epilepsy[MeSH Terms] AND (Infant[MeSH Terms] OR Child[MeSH Terms] OR Adolescent[MeSH Terms]) AND (Dietary Supplements[MeSH Terms] OR Infant Formula[MeSH Terms] OR prescrib*) | 22/02/2024 |
| Monitoring | (child* OR infant) AND (ketogenic OR "modified atkins") AND Epilepsy[MeSH Terms] AND ("monitoring" or "blood test" or "Monitoring, Ambulatory" or "lab*" or "blood work" or "adverse effects" [Subheading] or “side effects”) | 25/01/2024 |
| Adherence | (child* OR infant) AND (ketogenic OR “modified atkins”) AND Epilepsy[MeSH Terms] AND (psycho* OR social OR Treatment Adherence and Compliance[MeSH Terms] OR Patient Compliance[MeSH Terms]) | 13/02/2024 |
| Telemedicine | (child* OR infant) AND (ketogenic OR "modified atkins") AND Epilepsy[MeSH Terms] AND ( telemedicine* OR COVID-19 OR "virtual" OR "remote*" or "telehealth*") | 25/01/2024 |

**Table S2**: Components of modified ketogenic diets (% agreement rate from survey respondents)

| **‘Components’ of modified ketogenic diets** | **Agreement rate (survey respondents) *** |
| --- | --- |
| Specify carbohydrate limit and fat target, with protein allowed freely | 54% |
| Give all macro and kcal goals, including fat, carb and protein targets | 22% |
| Specify NET carbs only; calories and protein are free | 19% |
| I calculate the ratio of the diet, but only as a guide for myself | 18% |
| I calculate the diet using a ratio, but it is administered via household measurements and/or food exchanges | 17% |
| Specify TOTAL carbs only; calories and protein are free | 14% |
| Based on fat/protein/carb percentages | 11% |
| Other** | 9% |
| Ketogenic diet with a 2:1 or 1:1 ratio weighed on a gram scale | 6% |
| I don’t use MAD or MKD | 6% |
| Advise on a 50% total carb reduction | 3% |

* Respondents were able to select more than one answer

**‘Other’ comments:

1. Specify net carbohydrate, protein goal and fat goal
2. We give kcal, fat and carb goals
3. I give target macro ranges because most patients use a tracking app, but I tell patients that sticking to the carb limit is most important, they need to meet at least their minimum protein needs (OK to go over the provided range), and use fat as a tool to promote satiety and ketosis. People with a higher BMI may be able to eat less fat and use body stores. I tell patients to aim for at least 2 added fats per meal and at least one added fat per snack. Though I provide ranges, I’m flexible and teach patients how to think through building meals.
4. MAD = net carb restriction; MKD = ratio below 3:1 and measuring on gram scale
5. I also encourage fat and protein with every meal and snack.
6. We do different variations according to what suits the patient. We tend to give max carbs, min fat and a protein range. And will liberalize it from there if needed
7. I give minimum fat and maximum carbs in g and protein as a range in g. I give kcal as a range.
8. Specify net carb goal. Provide fat and protein targets.
9. I sometimes have to give protein goals
10. Like option 3 (limit CHO by grams, guide on rough portions of fat per meal to avoid hunger) but advise moderation/ caution with protein - based on their normal intake -and eat a portion at each meal. We don’t talk about any macro being free. Explain balance ...all macros influence ketosis.

**Table S3**: Carbohydrate allowances in initial modified ketogenic diet prescription for different age ranges (% agreement rate from survey respondents)

|  | **5g net** | **5g total** | **10g net** | **10g total** | **15g net** | **15g total** | **20g net** | **20g total** | **50% of baseline carb** | **5% energy from carb** | **10% energy from carb** | **15% energy from carb** | **Not Applicable** |
| --- | --- | --- | --- | --- | --- | --- | --- | --- | --- | --- | --- | --- | --- |
| **<2 yrs** | 2% | 3% | 9% | 10% | 5% | 1% | 7% | 6% | 2% | 3% | 5% | 3% | 21% |
| **2-5 yrs** | 0% | 1% | 9% | 13% | 9% | 5% | 7% | 5% | 2% | 1% | 6% | 5% | 16% |
| **6-11 yrs** | 0% | 0% | 6% | 6% | 13% | 8% | 13% | 4% | 4% | 2% | 4% | 6% | 13% |
| **12-18yrs** | 0% | 0% | 0% | 1% | 9% | 12% | 20% | 11% | 4% | 4% | 5% | 5% | 9% |

**Table S4**: List of commonly used ingredients and suggested alternatives for individuals with special dietary requirements

|  | **Dairy** | **Egg** | **Soy** | **Peanuts** | **Tree Nuts** | **Wheat/Gluten** |
| --- | --- | --- | --- | --- | --- | --- |
| **Ingredient** | **Substitute** | | | | | |
| Oil | ✔️ | ✔️ | ✔️ | ✔️ | ✔️ | ✔️ |
| Cream (Dairy) | Soy cream | ✔️ | Dairy cream | ✔️ | ✔️ | ✔️ |
| Egg | ✔️ | Raising agents  Baking soda  Baking powder  “No egg” substitute    Protein Source  Pea protein  Meat | ✔️ | ✔️ | ✔️ | ✔️ |
| Butter & Shortening | Olive oil butter  Almond butter  Coconut butter/shortening  Vegan butter,  Avocado | Olive oil butter  Almond butter  Coconut butter/shortening  Vegan butter,  Avocado | Dairy Butter  Almond butter  Vegan butter,  Avocado | Olive oil butter  Almond butter  Coconut butter/shortening  Vegan butter, Avocado | Olive oil butter  Vegan butter,  Avocado | Olive oil butter  Almond butter  Coconut butter/shortening  Vegan butter,  Avocado |
| Flour, Standard | Watch out for some Gluten free flour that contains dairy | Almond flour  Soy Flour  Walnut flour  Standard flour | Almond flour  Walnut flour  Standard flour | Almond flour  Soy Flour  Gluten Free flour  Walnut flour  Standard flour | Standard flour  Soy Flour  Gluten Free flour | Almond flour  Soy Flour  Gluten Free flour  Walnut flour |
| Generic Protein Powder | Pea protein | Skimmed milk powder  Protifar^TM^ | Skimmed milk powder  Protifar^TM^ | ✔️ | ✔️ | ✔️ |
| Generic Fat | Oil, dairy free butter/margarine | Oil, butter, avocado | Oil, butter, avocado | Oil, butter, avocado | Oil, butter, avocado | Oil, butter, avocado |
| Formula / milk | Plant based keto products  Rice milk  Almond milk  Soy milk | ✔️ | Rice milk  Almond milk  Dairy milk | ✔️ | ✔️ | ✔️ |
| Cheese | Soy cheese  Vegan cheese | ✔️ | Dairy cheese | ✔️ | ✔️ | ✔️ |
| Almond Flour | ✔️ | ✔️ | ✔️ | ✔️ | ✔️ | ✔️ |
| Yoghurt | Soy yoghurt | ✔️ | Dairy yoghurt | ✔️ | ✔️ | ✔️ |
| Avocado | ✔️ | ✔️ | ✔️ | ✔️ | ✔️ | ✔️ |
| Berries | ✔️ | ✔️ | ✔️ | ✔️ | ✔️ | ✔️ |
| Cocoa Powder | ✔️ | ✔️ | ✔️ | ✔️ | ✔️ | ✔️ |
| Cream Cheese | Soy cream cheese | ✔️ | ✔️ | ✔️ | ✔️ | ✔️ |

**Table S5**. Example classical ketogenic diet daily meal plan

| 3:1 ratio  9 year old child, Weight = 20 kg  Estimated dietary intake = 1339 kcal/day  3 meals and 2-3 snacks per day  *Gram measurements are rounded to 1 decimal place* | | | |
| --- | --- | --- | --- |
| ***Meal Option #1* *(Breakfast)* – Wheat Biscuit Cereal with Yoghurt** | | | |
|  | *Fat (g)* | *Protein (g)* | *Carbohydrate (g)*  *(excluding fibre)* |
| 5g Wheat biscuits (cereal) | 0.1 | 1.4 | 3.4 |
| 78g Unsweetened soya yoghurt | 1.1 | 4.4 | 0.6 |
| 38g 50% fat emulsion (or 20g oil) | 19 | - | - |
| 9g Blueberries, frozen | - | - | 1 |
| 20g Extra thick double or heavy cream | 10 | 0.3 | 0.2 |
| Water | - | - | - |
| ***Total*** | ***30.1*** | ***5.3*** | ***5.2*** |
| Directions:  Mix yoghurt and fat emulsion together with the extra thick cream. Top with crumbled wheat biscuits & berries.  *[Source of nutritional composition: New Zealand Food Composition Database (NZFCD). Values may vary between countries]* | | | |

| ***Meal Option #2* *(Lunch)* – Chickpea and Egg Curry** | | | |
| --- | --- | --- | --- |
|  | *Fat (g)* | *Protein (g)* | *Carbohydrate (g)*  *(excluding fibre)* |
| 90g Oil | *89.9* | *-* | *-* |
| 85g Mushrooms, raw, chopped | 0.2 | 2.0 | 0.8 |
| 50g Spinach, raw | 0.2 | 1.3 | 0 |
| 20g Spring onion, raw, chopped | 0.1 | 0.2 | 1.8 |
| 10g Tomato puree | 0 | 0.3 | 2.0 |
| 10g Curry powder | 1.1 | 0.9 | 3.7 |
| 5g Garlic, puree or minced | 0 | 0.4 | 0.4 |
| 80g Tinned chopped tomatoes | 0.1 | 1.0 | 2.6 |
| 52g Chickpeas, tinned, drained, no added salt | 0.8 | 3.6 | 7.7 |
| 80g Egg, cooked, chopped | 6.5 | 10.5 | 0.7 |
| 42g 50% fat emulsion | 21 | - | - |
| ***Total for 4 servings (batch)*** | **120** | **20** | **20** |
| ***Total for each serving*** | ***30*** | ***5*** | ***5*** |
| Directions:  Heat oil in a pan and add mushrooms. Allow the mushrooms to soak up the fat.  Add spinach, spring onion, tomato puree, curry paste and garlic puree.  Add chopped tomatoes and chickpeas and cook on low heat for 5 mins.  Let it simmer until the curry is slightly reduced.  Once vegetables are cooked, take off the heat and stir in the chopped egg and fat emulsion.  *[Source of nutritional composition: New Zealand Food Composition Database (NZFCD). Values may vary between countries]* | | | |

| ***Meal Option #3* *(Lunch)* – Stir Fry Konjac Noodles with Fried Egg and Capsicum** | | | |
| --- | --- | --- | --- |
|  | *Fat (g)* | *Protein (g)* | *Carbohydrate (g)*  *(excluding fibre)* |
| 3g Sesame seeds | 1.5 | 0.5 | 0.4 |
| 125g Konjac noodles | 1 | 1 | 1 |
| 36g Pepper, capsicum, yellow, raw or ¼ or ⅓ capsicum, chopped | 0.1 | 0.3 | 1.7 |
| 36g Pepper, capsicum, red, raw or ¼ capsicum, chopped | 0.1 | 0.3 | 1.6 |
| 21g Egg, whole, raw | 1.7 | 2.8 | 0.2 |
| 21g Vegetable Oil | 20.7 | 0 | 0.2 |
| 5g Sesame Oil | 5 | 0 | 0 |
| ***Total*** | ***30*** | ***5*** | ***5*** |
| Directions:  Drain the konjac noodles.  Beat the raw egg. Heat the pan with 5g vegetable oil. Fry the egg. Cut the fried egg into strips.  Use the same pan, heat the remaining 15g vegetable oil. Add onion and fry until golden and fragrant.  Add peppers, drained konjac noodles and soy sauce and stir fry.  Finally, add the egg strips. Cook and stir fry for a couple more minutes.  Top with sesame seeds and sesame oil.  *[Source of nutritional composition: McCance and Widdowson’s ‘The Composition of Foods seventh summary edition.’ CoFID 2015]* | | | |

| ***Meal Option #4* *(Dinner/Supper)* – Baked Cod with Mixed Vegetable ‘Couscous’** | | | |
| --- | --- | --- | --- |
|  | *Fat (g)* | *Protein (g)* | *Carbohydrate (g)*  *(excluding fibre)* |
| 10g Cod, baked | *0.1* | *2.1* | *0* |
| 25g Butter | 20.9 | 0.1 | 0.2 |
| 5g Lime juice | 0 | 0 | 0.1 |
| 4g Garlic puree | 0 | 0.3 | 0.4 |
| 50g Cauliflower, grated, cooked | 0.2 | 1.1 | 1.4 |
| 6.5g Olive oil | 6.5 | 0 | 0 |
| 30g Cherry tomatoes, chopped | 0.1 | 0.2 | 0.9 |
| 13g Green olives, pitted and chopped | 2 | 0.1 | 0 |
| 45g Courgette, chopped | 0.1 | 0.8 | 0.7 |
| 30g Red pepper, chopped | 0.1 | 0.3 | 1.3 |
| 1g Capers | 0 | 0 | 0 |
| ***Total*** | ***30*** | ***5*** | ***5*** |
| Directions:  In a small bowl, whisk together lime juice, melted butter, stir in the garlic and capers and season.  Steam or microwave the grated cauliflower and set aside.  Fry the chopped tomatoes, courgette and red pepper in olive oil. Once soft, combine with the cauliflower and green olives and add the butter mixture.  Serve with the baked cod.  *[Source of nutritional composition: McCance and Widdowson’s ‘The Composition of Foods seventh summary edition.’ CoFID 2015]* | | | |

| ***Snack Option #1 (Afternoon Tea)* – Chocolate Chip Cookie** | | | |
| --- | --- | --- | --- |
|  | *Fat (g)* | *Protein (g)* | *Carbohydrate (g)*  *(excluding fibre)* |
| 24g Butter | 19.5 | 0.2 | 0.2 |
| 75g Double or heavy cream (50% fat) | 36 | 1.3 | 1.9 |
| 70g Ground almonds/almond meal | 35.4 | 15.3 | 8.5 |
| 15g Sugar free chocolate chips | 4.4 | 1 | 3.6 |
| 40g Unsweetened desiccated coconut | 24.8 | 2.2 | 5.5 |
| 20g Stevia sweetener | - | - | - |
| ***Total for 8 servings (batch)*** | **120** | **20** | **20** |
| ***Total for each serving*** | **15** | **2.5** | **2.5** |
| Directions:  1) Preheat oven to 150 °C (300 °F).  2) Lightly grease a biscuit tray with oil spray.  3) Beat butter and sweetener together until pale and fluffy.  4) Mix all other ingredients together.  5) Using heaped teaspoon quantities, shape mixture into balls. If these do not form easily, add a little water to help combine them.  6) Place balls onto tray and flatten slightly with a fork.  7) Place in oven and bake for 20 - 25 mins.  *[Source of nutritional composition: New Zealand Food Composition Database (NZFCD) FOODFiles 2014. Values may vary between countries]* | | | |

| ***Snack Option #2 (Afternoon Tea)* – Chocolate Drink / Hot Chocolate** | | | |
| --- | --- | --- | --- |
|  | *Fat (g)* | *Protein (g)* | *Carbohydrate (g)*  *(excluding fibre)* |
| 10g Sugar free chocolate drinking powder | 0.1 | 0.2 | 0.8 |
| 26g Double or heavy cream (50% Fat) | 12.5 | 0.4 | 0.7 |
| 133g Unsweetened almond milk | 1.6 | 0.8 | 0.4 |
| 2.6g Sugar-free dark chocolate bits | 0.8 | 0.2 | 0.6 |
| 1g Protein modular | 0 | 0.9 | 0 |
| ***Total*** | **15** | **2.5** | **2.5** |
| Directions:  Put almond milk and cream in a small saucepan and cook over medium heat.  While the milk is heating, place the chopped chocolate in the microwave and heat for 30 seconds at half the power setting. Take the chocolate out and stir it. Return to the microwave and heat for another 30 seconds. Remove and stir. Continue to heat and stir the chocolate in 30 second intervals until melted, then stir in the chocolate drinking powder.  When the milk reaches the scalding point (bubbling around the edges), turn off the heat and whisk in the melted chocolate mixture.  *[Source of nutritional composition: New Zealand Food Composition Database (NZFCD) FOODFiles 2014. Values may vary between countries]* | | | |

| ***Snack Option #3 (Afternoon Tea)* – Vegetables and Cheese with Mayonnaise Platter** | | | |
| --- | --- | --- | --- |
|  | *Fat (g)* | *Protein (g)* | *Carbohydrate (g)*  *(excluding fibre)* |
| 5g Cheddar Cheese | 1.7 | 1.2 | 0.2 |
| 25g Cucumber with peel, raw | 0 | 0.2 | 0.4 |
| 15g Mayonnaise, Japanese style | 11.5 | 0.2 | 0.2 |
| 15g Olives, in brine | 1.6 | 0.1 | 0.4 |
| 15g Peas, frozen | 0.1 | 0.8 | 1.4 |
| ***Total*** | **15** | **2.5** | **2.5** |
| Directions:  Cut up ingredients into bite-size pieces. Arrange nicely like charcuterie board.  *[Source of nutritional composition: New Zealand Food Composition Database (NZFCD) FOODFiles 2014. Values may vary between countries]* | | | |

| ***Meal Option #5* - Blended Meal for Oral or Tube Feeding**  Dairy Free, Gluten Free, Soya Free, Egg Free, and Peanut Free | | | |
| --- | --- | --- | --- |
|  | *Fat (g)* | *Protein (g)* | *Carbohydrate (g)*  *(excluding fibre)* |
| 200g Lamb mince, raw | 40 | 33 | - |
| 175g Potato, raw, chopped | 0.1 | 3 | 34 |
| 150g Olive oil | 150 | - | - |
| 30g Peas, frozen | 0.2 | 1.5 | 3.2 |
| 50g Spinach, raw, chopped | 0.1 | 1 | 0.8 |
| 50g Broccoli, raw, chopped | 0.1 | 2 | 1.5 |
| 100g 50% fat emulsion | 50 | - | - |
| ***Total for 8 servings (batch)*** | **240.5** | **40.5** | **39.5** |
| ***Total for each serving*** | **30** | **5** | **4.9** |
| Directions:  Weigh and prepare all ingredients.  Heat 15g of olive oil in a pan and fry lamb mince until brown, then add the vegetables and some water.  Simmer for 10-15 mins (add more water if the mixture dries up quickly) until soft.  Turn off heat and allow to cool. Blend in the rest of the olive oil and emulsion.  Add water to get the desired consistency.  *[Source of nutritional composition: McCance and Widdowson’s ‘The Composition of Foods seventh summary edition.’ CoFID 2015]* | | | |

**Table S6**: Example modified ketogenic diet daily meal plan

| 9 year old child, Weight = 20 kg  Estimated dietary intake = 1339 kcal/day  3 meals and 2-3 snacks per day  *Gram measurements are rounded to 1 decimal place, as well as exact measurements* | | |
| --- | --- | --- |
| ***Meal Option #1 (Breakfast)* – Muesli with Yoghurt** | | |
|  | *Carbohydrate (g)(excluding fibre)* | *Fat (g)* |
| 3-4 tablespoons or ¼ cup (loosely packed) (31g) Brazil nuts (roughly 5-6 nuts) | 0 (0.1) | 21 (21.2) |
| ½ teaspoon (4g) Pumpkin seeds (shelled) | 0 (0.4) | 2 (1.7) |
| ~1 teaspoon (2g) Desiccated coconut (fine or medium texture) | 0 (0.1) | 1 (1.3) |
| ¼ teaspoon (1g) Cinnamon spice, ground | 0 (0.3) | 0 (0.03) |
| 1 teaspoon (2g) Rolled oats (loosely packed) | 1 (1.2) | 0 (0.1) |
| 1 teaspoon (5g) Coconut oil | 0 (0) | 5 (4.9) |
| ½ cup (120g) Low-carb yoghurt (1% carb) | 1 (0.96) | 2 (1.7) |
| ***Total*** | **3 (3.06)** | **31 (30.9)** |
| Directions:  Roughly chop Brazil nuts.  Fry nuts, pumpkin seeds, desiccated coconut, cinnamon, oats and half the coconut oil in a frying pan on a medium heat for approximately 5 minutes. Allow to cool.  Melt the second half of the coconut oil in a small ramekin in the microwave for approx 20-30 seconds.  Stir melted coconut oil into yoghurt.  Serve muesli with yoghurt mixture.  *[Source of nutritional composition: New Zealand Food Composition Database (NZFCD). Values may vary between countries]* | | |

| ***Meal Option #2 (Breakfast)* *–* Sour Milk with Myungu (Gourd)** | | |
| --- | --- | --- |
|  | *Carbohydrate (g)(excluding fibre)* | *Fat (g)* |
| 12g Myungyu, cooked | 1 (0.6) | 0 (0.03) |
| 50g Sour milk (fermented milk) | 2 (2.4) | 2 (1.5) |
| 26g Vegetable oil | 0 (0) | 26 (26) |
| 22g Avocado, Zambian | 0 (0.4) | 3 (3.4) |
| ***Total*** | **3 (3.4)** | **31 (30.9)** |
| Directions:  Mash the avocado, then add the gourd flesh and mix.  Add sour milk to the mixture. Add the oil and stir until evenly mixed.  *[Source of nutritional composition: Zambian food composition table, version 4. Values may vary between countries]* | | |

| ***Meal Option #3 (Breakfast)* *–* Scrambled Eggs, Bacon and Blueberries** | | |
| --- | --- | --- |
|  | *Carbohydrate (g)(excluding fibre)* | *Fat (g)* |
| 2 Medium slices bacon (42g) | 0 (0.21) | 12 (11.8) |
| 1 Small egg (45g) | 0 (0.4) | 4 (3.6) |
| 1 tablespoon (15g) Fresh cream (37–40% fat) | 0 (0.4) | 6g (5.5) |
| 2 teaspoons (10g) Olive oil | 0 (0) | 10 (9.9) |
| ¼ cup (16.5g) Blueberries (frozen) | 2 (1.9) | 0 (0.04) |
| ***Total*** | **3 (2.9)** | **31 (30.8)** |
| Directions:  Whisk egg and cream.  Heat pan with oil. Fry the egg mixture, slowly swirling it around the pan.  Grill or fry the bacon.  Serve scrambled eggs with bacon. Serve blueberries on the side.  *[Source of nutritional composition: New Zealand Food Composition Database (NZFCD). Values may vary between countries]* | | |

| ***Meal Option #4 (Lunch or Dinner)* *–* Bacon & Cheese Wrap** | |  |
| --- | --- | --- |
|  | *Carbohydrate (g)(excluding fibre)* | *Fat (g)* |
| 1 (30g) Low-carbohydrate wrap (4% carbs) | 2 (1.9) | 3 (3.3) |
| 1 tablespoon (15g) Mayonnaise | 0 (0.2) | 12 (11.8) |
| ¼ cup (30g) Cheddar cheese, grated | 0 (0.2) | 11 (10.6) |
| 3 slices (20g) Ham, thinly sliced | 0 (0.2) | 0 (0.42) |
| 1 tablespoon (2g) Carrot, raw, grated | 0 (0.1) | 0 (0) |
| ***Total*** | ***3 (2.9)*** | ***31 (31.1)*** |
| Directions:  Spread mayo over the wrap. Sprinkle cheese and carrot over the wrap and lay the ham over the top. Roll up wrap.  *[Source of nutritional composition: New Zealand Food Composition Database (NZFCD). Values may vary between countries]* | | |

| ***Meal option #5 (Lunch or Dinner)* *–* Avocado Salad** | | |
| --- | --- | --- |
|  | *Carbohydrate (g)(excluding fibre)* | *Fat (g)* |
| 1 small (or ½ to ⅔ medium) (116g) Avocado Fuerte variety, flesh  (NB: 100g California avocado has 1.52g carbs in 100g) *check your country’s food database* | 1 (0.7) | 26 (25.5) |
| 1 tablespoon (15g) Celery, chopped | 0 (0.2) | 0 (0.03) |
| 2 teaspoons (10g) Cream cheese, softened | 1 (0.5) | 4 (3.6) |
| 2 tablespoon (25g) Cucumber, diced | 0 (0.4) | 0 (0.04) |
| 2-3 (10g) or 1 tablespoon Olives, in brine, sliced | 0 (0.3) | 1 (1.1) |
| 2-3 (25g) Ham, thinly sliced (or 2 tablespoons Ham, diced) | 0 (0.3) | 1 (0.5) |
| 2 tablespoons (22g) Cherry tomatoes, chopped | 1 (0.7) | 0 (0.1) |
| ***Total*** | ***3 (3.1)*** | ***31 (30.9)*** |
| Directions:  Dice the avocado, celery, cucumber, tomatoes and ham into even pieces. Cut olives in half.  Mix all with the cream cheese to create a salad.  *[Source of nutritional composition: New Zealand Food Composition Database (NZFCD). Values may vary between countries]* | | |

| ***Meal option #6 (Lunch or Dinner)*** – **Moroccan Chicken** | | |
| --- | --- | --- |
|  | *Carbohydrate (g)*  *(excluding fibre)* | *Fat (g)* |
| 8-10 each (1kg) Chicken thighs, boneless | 0 (0) | 44 (43.6) |
| ¾ cup (200g) Olive oil | 0 (0.4) | 199 (199.2) |
| 2 teaspoons (10g) Garlic, crushed | 1 (0.9) | 0 (0.06) |
| 2 teaspoons (10g) Paprika | 2 (1.7) | 1 (0.7) |
| 1 teaspoon (4g) Cumin, ground, powder | 1 (1.4) | 1 (0.9) |
| ½ teaspoon (2g) Coriander ground, powder | 0 (0.4) | 1 (0.5) |
| ½ teaspoon (2g) Ginger ground, powder | 1 (1.3) | 0 (0.07) |
| 1 teaspoon (2.5g) Turmeric, ground, powder | 1 (1.1) | 0 (0.3) |
| 1 teaspoon (2.5g) Cinnamon, ground, powder | 1 (0.6) | 0 (0.08) |
| 1 can (400g) Tomatoes, diced | 13 (13.2) | 0 (0.4) |
| ¼ cup (30g) Chickpeas, tinned | 3 (3.1) | 0 (0.07) |
| ***Total for 8 servings (batch)*** | ***24 (24.1)*** | ***246 (257.9)*** |
| ***Total for each serving*** | ***3 (3)*** | ***31 (32.2)*** |
| Directions:  Mix all ingredients except chicken together in a small bowl to make a marinade.  Place chicken inside a zip-lock bag. Add marinade to the bag, press the air out and seal shut.  Rub marinade into the chicken until evenly coated.  Put the marinated chicken in the fridge for a few hours or overnight (optional).    Preheat oven to 180°C (350 °F).  Fry a few pieces of chicken at a time until lightly browned, then transfer to a baking dish. Add tinned tomatoes and chickpeas.  Cover and bake for 45 mins or until soft.  Suggestion: Serve with low/no carb rice or noodles.  *[Source of nutritional composition: New Zealand Food Composition Database (NZFCD). Values may vary between countries]* | | |

| ***Meal Option #7 (Lunch or Dinner) –* Sole Mediterranean** | | |
| --- | --- | --- |
|  | *Carbohydrate (g)(excluding fibre)* | *Fat (g)* |
| 8 small or 4-5 medium Fillets (850g) sole, fresh | 0 (0) | 10 (10.2) |
| 1 teaspoon (5g) Salt | 0 (0) | 0 (0) |
| 1 teaspoon (5g) Pepper | 0 (0) | 0 (0) |
| 2 teaspoon (4g) Cumin seeds, ground | 1 (1.4) | 1 (0.9) |
| 1 teaspoon (4g) Garlic powder | 3 (2.8) | 0 (0.03) |
| 3-4 each (15g) Garlic cloves, thinly sliced | 1 (1.3) | 0 (0.1) |
| 2 each Shallots or 2 tablespoons (20g) Shallots, thinly sliced | 2 (1.7) | 0 (0.02) |
| ½ cup (110g) Butter | 1 (1.1) | 117 (116.9) |
| ½ cup (120g) Olive oil | 0 (0) | 120 (119.5) |
| 1 tablespoon (20g) Lime juice | 0 (0.2) | 0 (0.04) |
| 2 tablespoons (10g) Capers | 0 (0.2) | 0 (0.05) |
| 8 (240g) Low-carbohydrate wraps (4% carbs) | 16 (15.6) | 26 (26.4) |
| Lemon slices and green onion, cut lengthways, for garnish |  |  |
| ***Total for 8 servings (batch)*** | ***24 (24.3)*** | ***274 (274.1)*** |
| ***Total for each serving*** | ***3 (3)*** | ***34 (34.3)*** |
| Directions:  In a small bowl, whisk together lime juice, olive oil and melted butter with a dash of seasoned salt.  Stir in the shallots, garlic and capers.  In a separate small bowl, mix together the seasoned salt, pepper, cumin and garlic powder. Spice fish fillets on both sides.  Place the fish fillets on a large lightly oiled baking pan or dish.  Cover with the buttery lime mixture.  Arrange the green onion and lemon slices on top (for flavour, not counted in the recipe).  Bake in oven at 190°C (375 °F) for 10-15 minutes.  Suggestion: Serve with a low carb wrap.  *[Source of nutritional composition: New Zealand Food Composition Database (NZFCD). Values may vary between countries]* | | |

| ***Meal option #8 (Lunch or Dinner) –* “Brazilian Feijoada”** | | |
| --- | --- | --- |
|  | *Carbohydrate (g)(excluding fibre)* | *Fat (g)* |
| ¼ cup or 2 tablespoons (50g) Black turtle beans, raw | 19 (18.5) | 1 (0.6) |
| 2 cups shredded/diced (300g) Jerk beef (roughly 10–12 slices, deli-style) | 9 | 48 |
| 1¼ cups of chopped Pork rib meat (without bone) (300g) (about 2–3 ribs) | 0 | 79 |
| 1¼ cups of diced Pork loin (300g) (roughly 1–2 pork loin chops) | 0 | 33 |
| 1⅓ cups diced or sliced (200g) Calabrese sausage | 3 | 60 |
| 1⅓ cups diced or sliced (200g) Paio sausage (half an average sausage) | 2 (1.7) | 46 |
| ½ cup (100g) chopped or diced Pork | 2 (1.6) | 62 (61.5) |
| 1 cup diced (200g) Bacon (approx. 7 rashers) | 1 (0.9) | 129 |
| For seasoning: |  |  |
| 1 cup chopped (150g) Onion, approximately 2 medium onions | 11 (10.7) | 0 (0.2) |
| 1 tablespoon minced (15g) Garlic (2.5 cloves) | 3 (3.4) | 0 |
| 2 Bay leaves | 0 | 0 |
| 3 tablespoons (45ml) Olive oil | 0 | 45 |
| Black pepper | 0 | 0 |
| Pinch fresh parsley | 0 | 0 |
| ***Total for 16 servings (batch)*** | ***49*** | ***502*** |
| ***Total for each serving*** | ***3*** | ***31*** |
| Directions:  Cook the black beans with the bay leaf in water until tender. Set aside.  In a small pan, brown the bacon, then add the de-salted meats. Once browned, add the sausages (calabrese and paio) and cook until all are nicely browned.  Add the cooked beans and their liquid to the pot with the meats. Simmer on a low heat for about 30 minutes. Add hot water if needed.  Heat the olive oil in a small skillet, then sauté the garlic and onion until golden. Add this to the feijoada, adjusting salt and pepper to taste.  Before serving, add freshly chopped herbs on top. Serve with konjac rice if desired.  *[Source of nutritional composition: Tabela Brasileira de Composição de Alimentos (TBCA). Universidade de São Paulo (USP). Food Research Center (FoRC). Version 7.2. São Paulo, 2023. Values may vary between countries]* | | |

| ***Meal Option #9 (Lunch or Dinner) –* Stir Fry Konjac Noodles with Fried Egg and Capsicum** | | |
| --- | --- | --- |
|  | *Carbohydrate (g)(excluding fibre)* | *Fat (g)* |
| 2.5ml Maggi Liquid Seasoning | 0 (0.2) | 0 (0) |
| ⅓ teaspoon (1g) Sesame seeds | 0 (0.2) | 1 (0.5) |
| 100g Konjac noodles (~1/2 packet) | 0 (0) | 0 (0) |
| ¼ cup (28g) chopped Pepper, capsicum, yellow, raw or ¼ to ⅓ capsicum | 1 (1.2) | 0 (0.1) |
| ¼ cup (28g) chopped Pepper, capsicum, red, raw or ¼ capsicum | 1 (1.3) | 0 (0.1) |
| ½ (30g) Egg, whole, raw | 0 (0.3) | 2 (2.4) |
| 1⅓ tablespoon (20g) Vegetable Oil | 0 (0.16) | 20 (19.8) |
| ½ tablespoon (8g) Sesame Oil | 0 (0) | 8 (7.9) |
| ***Total*** | ***3 (3.2)*** | ***31 (30.8)*** |
| Directions:  Drain the konjac noodles.  Beat the raw egg. Heat the pan with 5g vegetable oil. Fry the egg. Cut the fried egg into strips.  Use the same pan, heat the remaining 15g vegetable oil. Add onion and fry until golden and fragrant.  Add peppers, drained konjac noodles and soy sauce and stir fry.  Finally, add the egg strips. Cook and stir fry for a couple more minutes.  Top with sesame seeds and sesame oil.  *[Source of nutritional composition: New Zealand Food Composition Database (NZFCD). Values may vary between countries]* | | |

| ***Meal Option #10 (Lunch or Dinner)* *–* Fisashi** | |  |
| --- | --- | --- |
|  | *Carbohydrate (g)(excluding fibre)* | *Fat (g)* |
| 11g Chibwabwa (Pumpkin leaves) | 1 (0.77) | 0 (0) |
| 15g Groundnuts | 3 (2.55) | 7 (6.75) |
| 24g Vegetable Oil | 0 (0) | 24 (24) |
| ***Total*** | ***4 (3.32)*** | ***31 (30.75)*** |
| Directions:  Peel, dice and wash pumpkin leaves. Leave to boil for 5 minutes and add chopped tomatoes. Stir periodically for 10 minutes.  Add salt and groundnuts and leave to simmer until the groundnuts are completely submerged.  Cook for another 5 minutes, whilst stirring continuously.  *{Source of nutritional composition: Zambian food composition table, version 4. Values may vary between countries]* | | |

| ***Snack Option #1 –* Strawberry “Ice Cream”** | | |
| --- | --- | --- |
|  | *Carbohydrate (g)(excluding fibre)* | *Fat (g)* |
| 8-10 medium (100g) Strawberries (frozen) | 6 (5.5) | 0 (0.4) |
| ½ cup (95g) Double or heavy cream (45-48% fat) | 3 (2.5) | 46 (45.6) |
| 2 tablespoons (28g) 50% fat emulsion or 1 tablespoon (16g) vegetable oil | 0 (0) | 16 (16) |
| ***Total for 4 servings (batch)*** | **9 (8)** | **62 (62)** |
| ***Total for each serving*** | **2 (2)** | **15.5 (15.5)** |
| Add all ingredients to a food processor/blender and whizz to combine. You may need to scrape down the sides a few times between blasts. Add sweetener to taste.  Serve immediately for it to resemble soft-serve ice cream.  *[Source of nutritional composition: New Zealand Food Composition Database (NZFCD). Values may vary between countries]* | | |

| ***Snack Option #2 –* Bliss Balls or Fat Bombs** | | |
| --- | --- | --- |
|  | *Carbohydrate (g)(excluding fibre)* | *Fat (g)* |
| 1 tablespoon or 10-12 whole (15g) Almonds, roasted salted | 1 (1.1) | 8 (7.9) |
| 1 teaspoon (7.5g) Sugar free dark chocolate (chopped) | 2 (2.0) | 2 (2.4) |
| 1 tablespoon (15g) Cream cheese (softened) | 1 (0.8) | 6 (5.6) |
| ¼ cup (23.5g) Walnuts (4-5 whole walnuts) | 0 (0) | 15 (15.1) |
| ***Total for 2 servings (batch)*** | ***4 (3.9)*** | ***31 (31)*** |
| ***Total for each serving*** | ***2 (2)*** | ***15.5 (15.5)*** |
| Directions:  Roast almonds and walnuts in the oven until slightly brown, then crush them.  Mix the nuts together with all other ingredients.  Roll the mixture into balls and freeze for 20 mins before eating.  If left frozen for later, take ball(s) out of the freezer 10 mins before serving.  *[Source of nutritional composition: New Zealand Food Composition Database (NZFCD). Values may vary between countries]* | | |

| ***Snack Option #3 –* Chocolate Mousse** | | |
| --- | --- | --- |
|  | *Carbohydrate (g)(excluding fibre)* | *Fat (g)* |
| ½ cup mashed or ½ medium (120g) Avocado | 1 (0.7) | 26 (26.4) |
| 1 tablespoon (10g) Cocoa powder | 1 (0.9) | 2 (2.2) |
| 1 pottle or ½ cup (150g) Low-carb yoghurt (1% carb) | 1 (1.2) | 2 (2.1) |
| 2 tablespoons (50g) Stevia/ Nativia or 14.5g fibre maple syrup | 1 (1.1) | 0 |
| ***Total for 2 servings (batch)*** | ***4 (3.9)*** | ***30 (30.7)*** |
| ***Total for each serving*** | ***2 (2)*** | ***15 (15.4)*** |
| Directions:  Using a food processor, blend avocado, cocoa powder and sweetener until smooth.  Add yoghurt. Blend again until thick and creamy.  Divide mixture into 2 bowls or mugs. Serve immediately.  Suggestion: Use frozen avocado for a thicker consistency.  *[Source of nutritional composition: New Zealand Food Composition Database (NZFCD). Values may vary between countries]* | | |

| ***Snack Option #4 –* Keto Cheese Bread (Brazilian Pão de queijo)** | | |
| --- | --- | --- |
|  | *Carbohydrate (g)(excluding fibre)* | *Fat (g)* |
| 1 cup (100g) Almond flour | 9 (9) | 50 (49.9) |
| 1 ½ cups (150g) Parmesan cheese, grated | 7 (7.2) | 55 (55.1) |
| 1 Egg, whole (50g) | 1 (1.1) | 5 (4.5) |
| 2 tablespoons (30g) Melted butter | 0 (0.2) | 22 (22.3) |
| ¼ cup (60g) Cream cheese | 1 (1.2) | 13 (13.4) |
| ½ teaspoon (5g) Baking powder | 2 (2.2) | 0 (0) |
| Salt to taste (optional) | 0 (0) | 0 (0) |
| Black pepper (optional) | 0 (0) | 0 (0) |
| Garlic powder (optional) | 0 (0) | 0 (0) |
| ***Total for 10 servings (batch)*** | ***20 (20.9)*** | ***145 (145.2)*** |
| ***Total for each serving*** | ***2 (2.1)*** | ***15 (14.5)*** |
| Directions:  Preheat oven to 180°C (350°F).  Grease a baking tray or line with parchment paper.  In a large bowl, combine the almond flour, baking powder, salt, black pepper and garlic powder (if using).  Add the egg, melted butter, cream cheese and grated Parmesan cheese to the dry ingredients. Combine to make a smooth dough.  Grease your hands and shape the dough into small balls, about 25-30 g each (roughly the size of a walnut). Place the balls on the prepared baking tray, leaving some space between them.  Place in the oven and bake for 10-15 minutes, or until the cheese breads are golden brown and firm to the touch.  Leave to cool for a few minutes before serving.  *[Source of nutritional composition: Tabela Brasileira de Composição de Alimentos (TBCA). Universidade de São Paulo (USP). Food Research Center (FoRC). Version 7.2. São Paulo, 2023. Values may vary between countries]* | | |

**Table S7: 5g fat (long-chain triglyceride) choices for Modified ketogenic diets**

| Item | Weight (g) to provide 5g of fat | Household measurement to provide 5g fat* |
| --- | --- | --- |
| Butter | 6 | 1 teaspoon |
| Oil | 5 | 1 teaspoon |
| Mayonnaise | 7 | 1.5 teaspoon |
| Double cream | 10 | 2 teaspoons |
| Avocado | 33 | 2 tablespoons of mashed, or ¼ a medium avocado |

* rough approximations

**Table S8: 1g carbohydrate choices for Modified ketogenic diets**

| Item | Weight (g) to provide 1g carbohydrate | Household measurement to provide 1g carbohydrate* |
| --- | --- | --- |
| Apple, raw | 8 | One medium slice |
| Asparagus | 30 | 2-3 medium spears |
| Blueberries, raw | 7 | 4-5 medium-sized blueberries |
| Broccoli, raw | 31 | ½ cup of small florets |
| Cauliflower, raw | 23 | 2-3 small florets |
| Celeriac, raw | 43 | ⅓ cup of diced celeriac |
| Courgette, raw | 56 | ½ cup of chopped or sliced courgette |
| Fennel, raw | 50 | 1 cup of sliced fennel |
| Garlic, raw | 6 | 2-3 medium garlic cloves |
| Green leafy vegetables | 40-50 | 1 cup spinach, argula, or 2 cups lettuce |
| Onion, raw | 13 | 2 tablespoons of finely chopped onion |
| Peaches, raw | 13 | 1-2 slices |
| Raspberries, raw | 22 | 8-10 raspberries (medium-sized) |
| Strawberries, raw | 16 | 3-4 medium |
| Tomatoes, raw | 32 | 2-3 cherry tomatoes or ¼ of a medium tomato |
| Tomatoes, tinned | 26 | 2 tablespoons of drained, chopped tinned tomatoes |
| Tomatoes, puree | 8 | less than 1 tablespoon |

Ref: Matthew’s Friends choice lists, * rough approximations

**Table S9**: List of ketogenic prescribable products

| **Product** | **Company** | **Description** | **Indications*** |
| --- | --- | --- | --- |
| **Nutritionally complete** | | | |
| Ketocal 4:1 powder | Nutricia | Powdered feed enriched with fibre and LCPs available in vanilla and unflavoured varieties. The standard feed concentration is 14.3% | Suitable as a sole source of nutrition, or as a supplementary feed, in children over 1 year. For sip and tube feeding |
| Ketocal 4:1 LQ | Nutricia | Ready to use fibre enriched liquid feed available in vanilla and unflavoured varieties | Suitable as a sole source of nutrition in children aged 1–10 years or as a supplement for those over 10 years and adults |
| Ketocal 3:1 powder | Nutricia | Powdered feed, fibre‐free, enriched with LCPs, unflavoured. The standard feed concentration is 9.5% | Suitable as a sole source of nutrition in infants from birth to 6 years or as a supplement in those over 6 years (UK version). |
| Ketocal 2.5:1 LQ | Nutricia | Ready to use fibre‐enriched liquid feed available in vanilla | Suitable as a sole source of nutrition in children aged 8 years to adults or as a supplement |
| Ketovie 4:1 | Cortex Health / Ajinomoto Cambrooke Inc | Ready to use fibre, carnitine and citrate enriched liquid feed with 25% MCT. Available in unflavoured, vanilla or chocolate flavour | Suitable as a sole source of nutrition in children from 1 year of age |
| Ketovie Peptide 4:1 | Cortex Health / Ajinomoto Cambrooke Inc | Extensively hydrolyzed whey protein.  Ready to use fibre, carnitine and citrate enriched liquid feed with 15% MCT. | Suitable as a sole source of nutrition in children from 1 year of age |
| Ketovie 4:1 Plant-Based Protein | Cortex Health / Ajinomoto Cambrooke Inc | Pea protein  Ready to use fibre, carnitine and citrate enriched liquid feed with 25% MCT. | Milk and soy allergies.  Suitable as a sole source of nutrition in children from 1 year of age |
| Ketovie 3:1 | Cortex Health / Ajinomoto Cambrooke Inc | Ready to use partially hydrolyzed whey protein with 20% MCT.  Enriched with prebiotic fibre and carnitine. | Suitable as a sole source of nutrition in children from 1 year of age |
| K.Flo 4:1 | Nestle Health Science/Vitaflo | Ready to use fibre‐enriched liquid feed available in vanilla | Suitable from 3 years of age onwards |
| Ketonia | Namyang Dairy Products Co., Ltd. | Ready to use liquid formula for oral or enteral use in infants and young children | Suitable from birth |
| K.Yo | Nestle Health Science/Vitaflo | Ready to eat semi‐solid food | Suitable from 3 years of age onwards. Suitable as a sole source of nutrition up to 10 years of age |
| Ketobiota 2.5:1 | Dr Schaer/Kanso | Powdered, texture can be adapted (e.g. liquid or yogurt) with 60% MCT. Enriched with 11 vitamins | Suitable from 3 years of age onwards |
| KetoEpi 2:1 | Dr Schaer/Kanso | Ready to use liquid formula with 65% MCT, allergen-free | Suitable from 3 years of age onwards Suitable as a sole source of nutrition |
| KetVit | Dr Schaer/Kanso | Ready to eat creamy food, with 44% MCT. Enriched with minerals, vitamins and fiber  Ketogenic ratio 5.7:1 | Suitable from 3 years of age |
| MCTfiber | Dr Schaer/Kanso | Powder, 60% MCT and ketogenic ratio 7.2:1. Added with soluble fiber | Suitable from 3 years of age |
| DeliMCT creams (champignons, tomatoes, classical) | Dr Schaer/Kanso | Ready to use, enriched with MCT (ranging from 85% to 95%) | Suitable from 3 years of age |
| MCT Margarine 83% | Dr Schaer/Kanso | Ready to eat, with 83% MCT. Enriched with omega-3 + omega-6, vitamins A, D, E, folate, vitamin B12 | Suitable from 1 years of age  Maximum temperature 180° |
| DeliMCT Cacaobar | Dr Schaer/Kanso | Ready to eat, with 33% MCT, enriched in fiber. Ketogenic ratio 5.2:1 | Suitable from 3 years of age |
| KetoClassic 3:1 Bisk | Ketocare foods | 3:1 ratio high fat, high fibre food | Suitable from 3 years of age |
| KetoClassic 3:1 breakfast Porridge | Ketocare foods | 3:1 ratio, high fat, high fibre, ready prepared meal | Suitable from 3 years of age |
| KetoClassic 3:1 breakfast Muesli | Ketocare foods | 3:1 ratio, high fat, high fibre, ready prepared meal | Suitable from 3 years of age |
| KetoClassic 3:1 meal Savoury | Ketocare foods | 3:1 ratio, high fat, high fibre solid meal | Suitable from 3 years of age |
| KetoClassic 3:1 meal Chicken | Ketocare foods | 3:1 ratio, high fat, ready prepared meal | Suitable from 3 years of age |
| KetoClassic 3:1 meal Bolognese | Ketocare foods | 3:1 ratio, high fat, ready prepared meal | Suitable from 3 years of age |
| **Carbohydrate free formula** | | | |
| RCF | Abbott | Liquid feed very low in carbohydrate, soy protein | Milk allergy  Suitable from birth |
| Carb free mix | Nutricia | Powdered feed very low in carbohydrate | Suitable for infants and children |
| **Fat modules** | | | |
| Liquigen | Nutricia | 50% MCT emulsion | Suitable from birth |
| MCT oil | Nutricia | Liquid containing only a mixture of MCT | Suitable from birth |
| MCT oil (77% and 100%) | Dr Schaer/Kanso | Liquid containing only a mixture of MCT | Suitable from birth MCT 100% must be used raw |
| Calogen | Nutricia | 50% LCT fat emulsion | Suitable from birth |
| K.Quik | Nestle Health Science/Vitaflo | Ready to use 20% emulsion of MCT | Suitable from 3 years of age |
| **Protein modules** | | | |
| Protifar | Nutricia | Powdered milk based high protein supplement | Suitable from birth |
| Complete Amino Acid Mix | Nutricia | Powdered mix of essential and non-essential amino acids | Suitable from birth |
| Beneprotein | Nestle Health Science | Powdered milk based high protein supplement | Suitable from birth |
| MCT Procal | Nestle Health Science/Vitaflo | Neutral tasting protein  powder supplement high  in MCT | Suitable from 3 years of age |
| ProSource TF | Nutrinovo | Liquid high protein milk‐free (beef collagen derivative) supplement for tube feeding | Suitable from 3 years of age |
| **Carbohydrate modules** | | | |
| Polycal/Polyjoule | Nutricia | Powdered unflavoured carbohydrate supplement | Suitable from 1 year of age |
| Super Soluble Maxijul | Nutricia | Powdered neutral flavoured carbohydrate energy source | Suitable from birth |
| Vitajoule | Nestle Health Science/Vitaflo | Powdered unflavoured carbohydrate supplement | Suitable from birth |
| **Other** | | | |
| Keto Peptide | Functional Formularies | Whole foods-based formula (2.43:1 ratio). Includes peptide proteins. | No specific indications given |

LCP, long chain polyunsaturated fatty acids; MCT, medium chain triglycerides; LCT, long chain triglycerides

*Indications may vary between countries

**Table S10**: Example initiation schedules for an oral classical 3:1 ketogenic diet

| **Patient details:** 9 year old child, ‘Jamie.’ Appropriate growth history.  **Target CKD prescription 3:1 ratio**  Macronutrients: 1340 kcal, 20g protein, 130g fats and 23g carbohydrates |
| --- |

*Initiation over 2 weeks*

|  | Ratio increments in steps of 0.5 | Ratio increments in steps of 1 |
| --- | --- | --- |
| Day 1 | 1:1 (103g fat, 20g protein, 83g carbohydrate) | 1:1 (103g fat, 20g protein, 83g carbohydrate) |
| Day 2 |  |  |
| Day 3 |  |  |
| Day 4 | 1.5:1 (115g fat, 20g protein, 56.5g carbohydrate) |  |
| Day 5 |  |  |
| Day 6 |  |  |
| Day 7 | 2:1 (122g fat, 20g protein, 41g carbohydrate) | 2:1 (122g fat, 20g protein, 41g carbohydrate) |
| Day 8 |  |  |
| Day 9 |  |  |
| Day 10 | 2.5:1 (126g fat, 20g protein, 30g carbohydrate) |  |
| Day 11 |  |  |
| Day 12 |  |  |
| Day 13 | 3:1 (130g fat, 20g protein, 23g carbohydrate) |  |
| Day 14 |  | 3:1 (130g fat, 20g protein, 23g carbohydrate) |

*Initiation over 3 weeks*

|  | Ratio increments in steps of 0.5 | Ratio increments in steps of 1 |
| --- | --- | --- |
| Day 1 | 1:1 (103g fat, 20g protein, 83g carbohydrate) | 1:1 (103g fat, 20g protein, 83g carbohydrate) |
| Day 3 | 1.5:1 (115g fat, 20g protein, 56.5g carbohydrate) |  |
| Day 8 | 2:1 (122g fat, 20g protein, 41g carbohydrate) | 2:1 (122g fat, 20g protein, 41g carbohydrate) |
| Day 11 | 2.5:1 (126g fat, 20g protein, 30g carbohydrate) |  |
| Day 15 | 3:1 (130g fat, 20g protein, 23g carbohydrate) | 3:1 (130g fat, 20g protein, 23g carbohydrate) |

*Initiation over 6 weeks*

|  | Ratio increments in steps of 0.5 | Ratio increments in steps of 1 |
| --- | --- | --- |
| Week 1 | 0.5:1 (79g fat, 20g protein, 138g carbohydrate) | 1:1 (103g fat, 20g protein, 83g carbohydrate) |
| Week 2 | 1:1 (103g fat, 20g protein, 83g carbohydrate) |  |
| Week 3 | 1.5:1 1.5:1 (115g fat, 20g protein, 56.5g carbohydrate) | 2:1 (122g fat, 20g protein, 41g carbohydrate) |
| Week 4 | 2:1 (122g fat, 20g protein, 41g carbohydrate) |  |
| Week 5 | 2.5:1 (126g fat, 20g protein, 30g carbohydrate) |  |
| Week 6 | 3:1 (130g fat, 20g protein, 23g carbohydrate) | 3:1 (130g fat, 20g protein, 23g carbohydrate) |

**Table S11:** Example 3-week initiation schedule for an enteral classical 3:1 ketogenic diet

| **Patient details:** 9 year old child, ‘Jamie.’ Appropriate growth history.  **Target CKD prescription 3:1 ratio**  Macronutrients: 1340kcal, 20g protein, 130g fat and 23g carbohydrate  **5 feeds per day of 200ml each**  **Each feed to contain:** 268 kcal, 4g of protein, 26g of fat, 4.6g of carbohydrate |
| --- |

Week 1

|  | Fat (g) | Protein (g) | Carbohydrate (g) |
| --- | --- | --- | --- |
| 1:1 classical ratio (each feed: 20.6g fat, 4g protein, 16.6g carbohydrate) | | | |
| 16g carbohydrate polymer | - | - | 15.4 |
| 70ml water | - | - | - |
| 130ml 4:1 liquid keto formula (1.5kcal/ml) | 19.2 | 4 | 0.8 |
| 2ml 50% fat emulsion | 1 | - | - |
| **Total** | **20.2** | **4** | **16.2** |
| Mix the powder with the water then add liquid supplements | | | |

Week 2

|  | Fat (g) | Protein (g) | Carbohydrate (g) |
| --- | --- | --- | --- |
| 2:1 classical ratio (each feed: 24.4g fat, 4g protein, 8.2g carbohydrate) | | | |
| 8g carbohydrate polymer | - | - | 7.7 |
| 60ml water | - | - | - |
| 130ml 4:1 liquid keto formula (1.5kcal/ml) | 19.2 | 4 | 0.8 |
| 10ml 50% fat emulsion | 5 | - | - |
| **Total** | **24.2** | **4** | **8.5** |
| Mix the powder with the water then add liquid supplements | | | |

Week 3

|  | Fat (g) | Protein (g) | Carbohydrate (g) |
| --- | --- | --- | --- |
| 3:1 classical ratio (each feed: 26g fat, 4g protein, 4.6g carbohydrate) | | | |
| 4g carbohydrate polymer | - | - | 3.8 |
| 50ml water | - | - | - |
| 130ml 4:1 liquid keto formula (1.5kcal/ml) | 19.2 | 4 | 0.8 |
| 14ml 50% fat emulsion | 7 | - | - |
| **Total** | **26.2** | **4** | **4.6** |
| Mix the powder with the water then add liquid supplements | | | |

**Table S12**. Example initiation schedule for a modified ketogenic diet

| Patient details: 9 year old ‘Jamie’ | | MKD prescription:  **104g/day FAT (31g/meal and 15.5g/snack)**  **15g/day CARBOHYDRATE (3g/meal and 2g/snack)**  3 meals and 3-4 snacks per day |
| --- | --- | --- |
| **Option 1: advance by macronutrient prescription**  *Full calories, no fasting period* | | |
| **Week 1** | Start with 40% energy from fat and 50g carb/day [starting amounts can be agreed with the individual/family]  **Fat:** 40% x 1339 kcal = 535.6 kcal ÷ 9kcals = **59.5 g fat per day**  **(6.5g/snack and 13g/meal)**  **Carbohydrate: 50g/day (6-7g/snack and 10g/meal)**  Protein food/portions remain as before (or age-appropriate portions) | |
| **Week 2** | 50% energy from fat and 30g carb/day  **Fat:** 50% x 1339 kcal =669.5 kcal ÷ 9kcals = **74.4g fat per day**  **(8g/snack and 16.5g/meal)**    **Carbohydrate: 30g/day (2g/snack and 8g/meal)**  Protein food/portions remain as before (or age-appropriate portions) | |
| **Week 3** | 60% energy from fat and 20g carb/day  **Fat:** 60% x 1339 kcal = 803.4 kcal ÷ 9kcals = **89.3g fat per day**  **(10g/snack and 20g/meal)**  **Carbohydrate: 20g/day (1-2g/snack and 5g/meal)**  Protein food/portions remain as before (or age-appropriate portions) | |
| **Week 4** | 70% energy from fat and 10g carb/day  **Fat:** 70% x 1339 kcal = 937.3 kcal ÷ 9kcals = **104g fat per day**  **(15.5g/snack and 31g/meal)**  **Carbohydrate: 10g/day (1g/snack and 2g/meal)**  Protein food/portions remain as before (or age-appropriate portions) | |
| **Option 2: add in one ketogenic meal at a time** | | |
| **Week 1:**  **MKD Lunch** | Breakfast: as usual  Morning snack: as usual  **Lunch: MKD (31g Fat (6 choices), 3g Carb (3 choices))**  Afternoon Snack: as usual  Dinner: as usual  Bedtime snack: as usual | |
| **Week 2:**  **MKD Breakfast + lunch** | **Breakfast:** **MKD (31g Fat (6 choices), 3g Carb (3 choices))**  Morning snack: as usual  **Lunch: MKD (31g Fat (6 choices), 3g Carb (3 choices))**  Afternoon Snack: as usual  Dinner: as usual  Bedtime snack: as usual | |
| **Week 3:**  **MKD Breakfast + lunch + dinner** | **Breakfast:** **MKD (31g Fat (6 choices), 3g Carb (3 choices))**  Morning snack: as usual  **Lunch: MKD (31g Fat (6 choices) 3g Carb (3 choices))**  Afternoon Snack: as usual  **Dinner:** **MKD (31g Fat (6 choices), 3g Carb (3 choices))**  Bedtime snack: as usual | |
| **Week 4:**  **MKD for all meals and snacks** | **Breakfast: MKD (31g Fat (6 choices), 3g Carb (3 choices))**  **Morning snack: MKD (15.5g Fat (3 choices), 2g Carb (2 choice))**  **Lunch: MKD (31g Fat (6 choices), 2g Carb (2 choices))**  **Afternoon Snack: MKD (15.5g Fat (3 choices), 2g Carb (2 choice))**  **Dinner: MKD (31g Fat (6 choices), 3g Carb (3 choices))**  **Supper: MKD (15.5g Fat (3 choices), 2g Carb (2 choice))** | |

|  |
| --- |

**Table S13**. Advantages and disadvantages of the use of telemedicine for ketogenic diet therapy

| **Advantages** | **Disadvantages** |
| --- | --- |
| Reduces travel time, so particularly beneficial for patients/families who have to travel long distances, potentially with nursing needs such as suction difficulties or mechanical ventilation, and/or behavioral disorders or mobility issues (1)  Environmental benefit of reducing travel (2) | May not be appropriate for young infants, clinically unstable patients, those at high risk of hypoglycemia or metabolic issues, families without access to technology, and those unable to access emergency medical care if necessary (3) |
| Can reduce waiting lists (1) | Inconsistency with anthropometric measurements and difficulties in getting clinical information/results (labs and other routine assessments) (1, 3) |
| May reduce patient / family stress (2) | Detailed discussions may seem less personal when done by video call - particularly pertinent when discussing diet discontinuation, or lack of response to treatment (1, 3) |
| Multiple family members and caregivers may be able to join the education sessions (3) | Technological aspects, such as difficulties with connecting online, can be a limiting factor (1) |
| Allows the team to see the patient in their own house (potentially including the kitchen) (2) |  |
| Electronic communication may be appealing for adolescents (4) |  |

References for Table S13:

1. Armeno M, Caballero E, Verini A*, et al.* Telemedicine- versus outpatient-based initiation and management of ketogenic diet therapy in children with drug-resistant epilepsy during the COVID-19 pandemic. *Seizure*. 2022, **98** 37-43.

2. Bara VB, Schoeler N, Carroll JH*, et al.* Patient and carer perspectives on the use of video consultations in the management of the ketogenic diet for epilepsy. *Epilepsy Behav*. 2023, **145** 109280.

3. Kossoff EH, Turner Z, Adams J*, et al.* Ketogenic diet therapy provision in the COVID-19 pandemic: Dual-center experience and recommendations. *Epilepsy Behav*. 2020, **111** 107181.

4. Cervenka MC, Henry BJ, Felton EA*, et al.* Establishing an Adult Epilepsy Diet Center: Experience, efficacy and challenges. *Epilepsy Behav*. 2016, **58** 61-8.

**Table S14**. Worked examples of how to discontinue a classical ketogenic diet

| **Patient details:**  9-year-old child, ‘Jamie.’ Appropriate growth history. 3 meals and 3 snacks per day for an oral CKD or 6 equal feeds for enteral CKD  Short time on the diet (less than 3 months) and/or need to rapidly wean off the diet  Full CKD prescription **3:1 ratio**  1340,00 kcal, 20 g proteins, 129.7 g fats and 23.2 g carbohydrates  **3 meals and 3 snacks a day** | |
| --- | --- |
| **One-week discontinuation** protocol with one CKD ratio decrease every 2-3 days* | |
| Days 1-3 | **2:1 ratio**  1340 kcal, 20 g protein, 121.8 g fats and 40.9 g carbohydrates |
| Days 4-6 | **1:1 ratio**  1340 kcal, 20 g protein, 103.1 g fats and 83.1 g carbohydrates |
| Day 7 | **Liberal/usual diet** |
| **One-week discontinuation** protocol with a 0.5 CKD ratio decrease every 2 days | |
| Day 1-2 | **2.5:1 ratio**  1340 kcal, 20 g protein, 126.5 g fats and 30.6g carbohydrates |
| Day 3-4 | **2:1 ratio**  1340 kcal, 20 g protein, 121.8 g fats and 40.9 g carbohydrates |
| Day 5-6 | **1.5:1 ratio**  1340 kcal, 20 g protein, 115 g fats and 56.5 g carbohydrates |
| Day 7 | **1:1 ratio**  1340 kcal, 20 g protein, 103.1 g fats and 83.1 g carbohydrates |
| Day 8 | **Liberal/usual diet**  ***If the initial prescription ratio is 4:1, you can decrease by one CKD ratio every 2 days** |
| **Six-weeks discontinuation** protocol with one CKD ratio decrease every 2 weeks | |
| **Weeks 1-2** | **2:1 ratio**  1340 kcal, 20 g protein, 121.8 g fats and 40.9 g carbohydrates |
| **Weeks 3-4** | **1:1 ratio** 1340 kcal, 20 g protein, 103.1 g fats and 83.1 g carbohydrates |
| **Week 5** | **Liberal/usual diet** |
| **Four-weeks discontinuation** protocol with a 0.5 CKD ratio decrease every week** | |
| **Week 1** | **2.5:1 ratio**  1340 kcal, 20 g protein, 126.5 g fats and 30.6g carbohydrates |
| **Week 2** | **2:1 ratio**  1340 kcal, 20 g protein, 121.8 g fats and 40.9 g carbohydrates |
| **Week 3** | **1.5:1 ratio**  1340 kcal, 20 g protein, 115 g fats and 56.5 g carbohydrates |
| **Week 4** | **1:1 ratio**  1340 kcal, 20 g protein, 103.1 g fats and 83.1 g carbohydrates |
| **Week 5** | **Liberal/usual diet**  **** If the initial prescription is 4:1 CKD ratio, the same can be done in 4 weeks with a one CKD ratio decrease each 7 days** |

**Table S15**. Worked examples of how to discontinue an enteral classical ketogenic diet.

| **Patient details:** 9 year old child, ‘Jamie.’ Appropriate growth history.  **3:1 ratio**  **6 feeds per day** |
| --- |
| **3:1 ratio**  1340 kcal, 20 g protein, 129.7 g fats and 23.2g carbohydrates  **Target content for each feed:** 233 kcal, 3.3 g of protein, 21.6 g of fats, 3.9 g of carbohydrates   \|  \| Protein (g) \| Fat (g) \| Carbohydrate (g) \| \| --- \| --- \| --- \| --- \| \| 107ml 4:1 ketogenic formula \| 3.3 \| 15.8 \| 0.7 \| \| 3.2g Carbohydrate module \| 0 \| 0 \| 3 \| \| 11.6ml 50% fat emulsion module \| 0 \| 5.8 \| 0.0 \| \| **Total** \| **3.3** \| **21.6** \| **3.9** \| |
| **Step 1 - week 1**  **2:1 ratio**  1340 kcal, 20 g protein, 121.8 g fats and 40.9 g carbohydrates  **Target content for each feed:** 233 kcal, 3.3 g of protein, 20.3 g of fats, 6.8 g of carbohydrates   \|  \| Protein (g) \| Fat (g) \| Carbohydrate (g) \| \| --- \| --- \| --- \| --- \| \| 80ml 4:1 ketogenic formula \| 2.5 \| 11.8 \| 0.5 \| \| 55ml 1kcal/ml enteral formula \| 1.5 \| 2.7 \| 6.3 \| \| 13ml 50% fat emulsion module \| 0 \| 6.5 \| 0.0 \| \| **Total** \| **4** \| **21** \| **6.8** \| |
| **Step 2 - week 2**  **1:1 ratio**  Daily total 1340kcal, 20 g protein, 103.1 g fats and 83.1 g carbohydrates  **Target content for each feed:** 233 kcal, 3.3 g of protein, 17.2 g of fats, 13.9 g of carbohydrates   \|  \| Protein (g) \| Fat (g) \| Carbohydrate (g) \| \| --- \| --- \| --- \| --- \| \| 30ml 4:1 ketogenic formula \| 0.6 \| 2.8 \| 0.1 \| \| 110ml 1kcal/ml enteral formula \| 2.8 \| 4.4 \| 13.8 \| \| 20ml 50% fat emulsion module \| 0 \| 10 \| 0.0 \| \| **Total** \| **3.3** \| **17.2** \| **13.9** \|   Then transition to normal enteral formula |

**Table S16.** Worked examples of how to discontinue a modified ketogenic diet

| **SCENARIO 1**  ‘Jamie’ has been on diet for approximately 3 months and is discontinuing due to no effect on seizures.  MKD prescription:  **104g/day FAT (31g/meal and 15.5g/snack)**  **10g/day CARBOHYDRATE (3g/meal and 2g/snack)**  3 meals and 3 snacks per day  **ONE WEEK DISCONTINUATION PROTOCOL** | |
| --- | --- |
| **Option 1: advance by macronutrient prescription** | |
| Day 1 | **65% fat and 30g carbohydrate**  **Fat:** 65% x 1339 kcal = 870.4 kcal ÷ 9kcals = **96.7g fat per day (23g/meal and 9g/snack)**  **Carbohydrate: 30 g/day (7g/meal and 3g/snack)**  Protein food/portions remain as before (or age-appropriate portions) |
| Day 2 | **60% fat and 40g carbohydrate**  **Fat:** 60% x 1339 kcal = 803.4 kcal ÷ 9kcals = **89.3g fat per day (22g/meal and 7g/snack)**  **Carbohydrate: 40 g/day (9g/meal and 4g/snack)**  Protein food/portions remain as before (or age-appropriate portions) |
| Day 3 | **55% fat and 50 g carbohydrate**  **Fat:** 55% x 1339 kcal = 736.5 kcal ÷ 9kcals = **81.8g fat per day (22g/meal and 5g/snack)**  **Carbohydrate: 50 g/day (13g/meal and 7g/snack)**  Protein food/portions remain as before (or age-appropriate portions) |
| Day 4 | **50% fat and 60g carbohydrate**    **Fat:** 50% x 1339 kcal = 669.5 kcal ÷ 9kcals = **74.4g fat per day (20g/meal and 4g/snack)**  **Carbohydrate: 60 g/day (13g/meal and 7g/snack)**  Protein food/portions remain as before (or age-appropriate portions) |
| Day 5 | **45% fat and 80 g carbohydrate**  45% x 1339 kcal = 602.6 kcal ÷ 9kcals = **67g fat per day (19g/meal and 3g/snack)**  **Carbohydrate: 80 g/day (18g/meal and 9g/snack)**  Protein food/portions remain as before (or age-appropriate portions) |
| Day 6 | **5% fat and 100g carbohydrate**  45% x 1339 kcal = 602.6 kcal ÷ 9kcals = **67g fat per day (19g/meal and 3g/snack)**  **Carbohydrate: 100 g/day (22g/meal and 11g/snack)**  Protein food/portions remain as before (or age-appropriate portions) |
| Day 7 | **Usual diet** |
|  | |
| **Option 2: wean off one MKD meal at a time** | |
| Day 1 | Breakfast: MKD  Morning snack: MKD  **Lunch: no restrictions / usual (pre-MKD) meal**  Afternoon snack: MKD  Dinner: MKD  Bedtime snack: MKD |
| Day 2 | Breakfast: MKD  Morning snack: MKD  **Lunch: no restrictions / usual (pre-MKD) meal**  Afternoon snack: MKD  Dinner: MKD  **Bedtime snack:** **no restrictions / usual (pre-MKD) meal** |
| Day 3 | **Breakfast:** **no restrictions / usual (pre-MKD) meal**  Morning snack: MKD  **Lunch: no restrictions / usual (pre-MKD) meal**  Afternoon snack: MKD  Dinner: MKD  **Bedtime snack:** **no restrictions / usual (pre-MKD) meal** |
| Day 4 | **Breakfast:** **no restrictions / usual (pre-MKD) meal**  Morning snack: MKD  **Lunch: no restrictions / usual (pre-MKD) meal**  Afternoon snack: MKD  **Dinner:** **no restrictions / usual (pre-MKD) meal**  **Bedtime snack: no restrictions / usual (pre-MKD) meal** |
| Day 5 | **Breakfast:** **no restrictions / usual (pre-MKD) meal**  **Morning snack**: **no restrictions / usual (pre-MKD) snack**  **Lunch: no restrictions / usual (pre-MKD) meal**  Afternoon Snack: MKD  **Dinner: no restrictions / usual (pre-MKD) meal**  **Bedtime snack: no restrictions / usual (pre-MKD) meal** |
| Days 6-7 | **All meals / snacks – no restrictions / usual (pre-MKD) diet** |
|  | |
| **SCENARIO 2**  ‘Jamie’ has been on diet, with good response, for more than 2 years. He is discontinuing the diet as he has been on it for over 2 years.  MKD prescription:  **104g/day FAT (31g/meal and 15.5g/snack)**  **15g/day CARBOHYDRATE (3g/meal and 2g/snack)**  3 meals and 3 snacks per day  **FOUR WEEK DISCONTINUATION PROTOCOL** | |
| **Option 1: advance by macronutrient prescription*** | |
| Week 1 | **65% fat and 20g carbohydrate**  **Fat:** 65% x 1339 kcal = 870.4 kcal ÷ 9kcals = **96.7g fat per day (23g/meal and 9g/snack)**  **Carbohydrate: 20 g/day (4g/meal and 2g/snack)**  Protein food/portions remain as before (or age-appropriate portions) |
| Week 2 | **55% fat and 25g carbohydrate**    **Fat:** 55% x 1339 kcal = 736.5 kcal ÷ 9kcals = **81.8g fat per day (22g/meal and 5g/snack)**  **Carbohydrate: 25 g/day (3g/meal and 5g/snack)**  Protein food/portions remain as before (or age-appropriate portions) |
| Week 3 | **45% fat and 30g carbohydrate**  45% x 1339 kcal = 602.6 kcal ÷ 9kcals = **67g fat per day (19g/meal and 3g/snack)**  **Carbohydrate: 30 g/day (7g/meal and 3g/snack)**    Protein food/portions remain as before (or age-appropriate portions) |
| Week 4 | **35% fat and 40g of carbohydrate**  35% x 1339 kcal = 602.6 kcal ÷ 9kcals = **52g fat per day (14g/meal and 3g/snack)**  **Carbohydrate: 40 g/day (9g/meal and 4g/snack)**  Protein food/portions remain as before (or age-appropriate portions) |
| Week 5 | **35% fat and 50g of carbohydrate**  35% x 1339 kcal = 602.6 kcal ÷ 9kcals = **52g fat per day (14g/meal and 3g/snack)**  **Carbohydrate: 50 g/day (11g/meal and 5.5g/snack)**  Protein food/portions remain as before (or age-appropriate portions |
| Week 6 | **35% fat and 60g of carbohydrate**  35% x 1339 kcal = 602.6 kcal ÷ 9kcals = **52g fat per day (14g/meal and 3g/snack)**  **Carbohydrate: 60 g/day (13g/meal and 7g/snack)**  Protein food/portions remain as before (or age-appropriate portions) |
| Week 7 | **35% fat and 70g of carbohydrate**  35% x 1339 kcal = 602.6 kcal ÷ 9kcals = **52g fat per day (14g/meal and 3g/snack)**  **Carbohydrate: 70 g/day (15.5g/meal and 8g/snack)**  Protein food/portions remain as before (or age-appropriate portions) |
| Week 8 | **35% fat and 80g of carbohydrate**  35% x 1339 kcal = 602.6 kcal ÷ 9kcals = **52g fat per day (14g/meal and 3g/snack)**  **Carbohydrate: 80 g/day (18g/meal and 9g/snack)**  Protein food/portions remain as before (or age-appropriate portions) |
| Week 9 | **Usual diet** |
|  | |
| **Option 2: wean off one MKD meal at a time** | |
| Week 1 | Breakfast: MKD  Morning snack: MKD  **Lunch: no restrictions / usual (pre-MKD) meal**  Afternoon snack: MKD  Dinner: MKD  Bedtime snack: MKD |
| Week 2 | Breakfast: MKD  Morning snack: MKD  **Lunch: no restrictions / usual (pre-MKD) meal**  Afternoon snack: MKD  Dinner: MKD  **Bedtime snack:** **no restrictions / usual (pre-MKD) meal** |
| Week 3 | **Breakfast:** **no restrictions / usual (pre-MKD) meal**  Morning snack: MKD  **Lunch: no restrictions / usual (pre-MKD) meal**  Afternoon snack: MKD  Dinner: MKD  **Bedtime snack:** **no restrictions / usual (pre-MKD) meal** |
| Week 4 | **Breakfast:** **no restrictions / usual (pre-MKD) meal**  Morning snack: MKD  **Lunch: no restrictions / usual (pre-MKD) meal**  Afternoon snack: MKD  **Dinner:** **no restrictions / usual (pre-MKD) meal**  **Bedtime snack: no restrictions / usual (pre-MKD) meal** |
| Week 5 | **All meals / snacks – no restrictions / usual (pre-MKD) diet** |

*this discontinuation protocol is based on the re-introduction of 20 g carbohydrates per week and 10% decrease in fat per week. This is an example only – the amount of carbohydrate and fat that is changed per unit of time is to be decided by the dietitian and will partly depend on the initial MKD prescription
